# Non-targeted metabolomics-mediated elucidation of metabolite changes in *Polygonatum kingianum* during traditional steaming with black beans

**DOI:** 10.3389/fnut.2025.1581459

**Published:** 2025-04-11

**Authors:** Haiyan Zhu, Zaoxia Pei, Lei Zhang, Jiahong Dong, Pengzhang Ji

**Affiliations:** ^1^Yunnan Key Laboratory of Southern Medicinal Resource, School of Chinese Materia Medica, Yunnan University of Chinese Medicine, Kunming, China; ^2^Institute of Medicinal Plant Cultivation, Academy of Southern Medicine, Yunnan University of Chinese Medicine, Kunming, China

**Keywords:** *Polygonatum kingianum*, black bean, metabolomics, organoleptic evaluation, nine steaming and drying cycles

## Abstract

**Introduction:**

*Polygonatum* is a traditional medicinal and edible plant often prepared by steaming it with ingredients like yellow rice wine and black beans. However, the metabolic changes in *Polygonatum kingianum* (PK) during the traditional steaming method with black beans have not been previously reported. This study aims to explore how the addition of black beans and the degree of steaming affect the quality of PK.

**Methods:**

This study quantified polysaccharides, reducing sugars, saponins, and flavonoids in PK subjected to varied steaming cycles, while also integrating color measurement, sensory evaluation, and non-targeted metabolomics analysis.

**Results:**

PK is rich in lipids, amino acids, sugars, flavonoids, and organic acids. Notably, we observed the production and significant accumulation of daidzein and daidzin (two primary active compounds derived from black beans) as well as triterpenoid saponins (including Sandosaponin A, Soyasaponin II-III, V, and Pisumsaponin I-II) in steamed PK (*p* < 0.05). After three steaming cycles, multiple bioactive compounds levels peaked or stabilized, which was consistent with the sensory quality (score: 94/100). Compared to the raw PK, the P3 stage exhibited significant increases in saponins, total sugars, organic acids, and flavonoids by 21.29, 35.02, 49.13, and 44.76%, respectively (*p* < 0.05), reducing sugars showed a remarkable increase of 160.94-fold (*p* < 0.01), while polysaccharides decreased significantly by 43.33% (*p* < 0.01).

**Conclusion:**

This study provides the first evidence that steaming with black beans can synergistically increase the diversity of triterpenoid saponins and isoflavones in PK, with the optimal quality achieved after three rounds of steaming. These findings validate the scientific efficacy of the traditional processing method. Our results advocate for the continued use of the black bean steaming method to enhance the nutritional and health-promoting value of PK, while also providing a template for the innovation of herbal processing techniques based on auxiliary ingredients.

## Graphical abstract

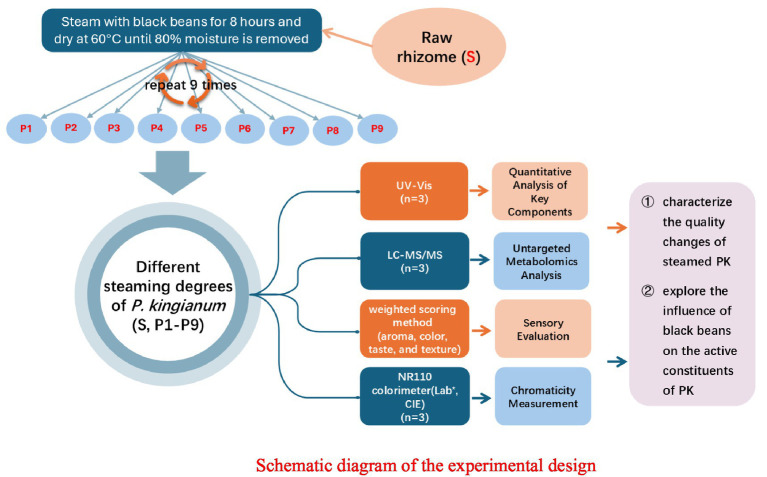

## 1 Introduction

*Polygonatum* is a traditional Chinese medicinal and edible plant, with its rhizomes commonly used in medicine. It was first mentioned in the *Miscellaneous Records of Famous Physicians (Ming Yi Bie Lu)*, where it is noted that “long-term use will lighten the body, prolong life, and prevent hunger.” The main medicinal species of *Polygonatum* listed in the *Chinese Pharmacopoeia* include *Polygonatum kingianum* Coll. et Hemsl. (PK), *Polygonatum sibiricum* Red., and *Polygonatum cyrtonema* Hua. PK, primarily cultivated in Yunnan Province, has garnered increasing attention for its high yield, medicinal properties, and nutritional value. Recent studies have demonstrated that PK exhibits a range of physiological activities, including anti-fatigueeffects (1), antioxidant properties (2), anti-inflammatory action (3), anti-aging benefits (4), hypolipidemic effects (5), hypoglycemic effects (6).

Raw *Polygonatum* is often processed before use, as it can be irritating and cause allergic reactions in its unprocessed form (7). Recently, increasing research has focused on the effects of steaming on the intrinsic components of *Polygonatum.* Research has found that the steaming time significantly influences the digestion, absorption, and promotion of gut microbiota fermentation of polysaccharides in *P. cyrtonema* (8). Jin et al.’s study (9) demonstrated that during the steaming of *P. cyrtonema* rhizomes, polysaccharides and oligosaccharides gradually break down into monosaccharides, such as glucose, galactose, and fructose. They noted that fructose can be used as a marker for the steaming process of these rhizomes. Meanwhile, a study has indicated that fructan serves as the principal carbohydrate component in raw *Polygonatum*. During the steaming process, fructan undergoes significant degradation, with complete degradation observed after seven steaming cycles. This suggests that the method involving nine steaming cycles may lead to over-processing (10). In a study investigating the changes in secondary metabolites during the steaming process of *P. cyrtonema*, it was found that the first three steaming cycles significantly promoted the accumulation of functional components, while subsequent cycles led to varying degrees of degradation of these components. Based on these findings, the authors recommend reducing the traditional nine steaming cycles to a range of 3–6 cycles (11). Zhu et al. (12) examined the effects of nine steaming treatments combined with various drying methods on multiple components of *P. cyrtonema* rhizomes. They observed that chemical components stabilized after four steamings, achieving an optimal taste and flavor. They suggested that a four-steaming process combined with hot-air drying may be a viable alternative to the traditional method of nine steaming cycles. Another study has indicated that total flavonoids and homoisoflavonoids in *P. cyrtonema* increase significantly with prolonged steaming, while their antioxidant and hypoglycemic activities are also markedly enhanced (13).

Besides the conventional methods of steaming and the nine steaming cycles (repeating the steaming and drying process nine times to reduce toxicity or enhance the therapeutic effect), the processing of *Polygonatum* often incorporates auxiliary materials to further enhance its medicinal effect. Common auxiliary materials include yellow rice wine, black beans, honey, cooked *Rehmannia glutinosa*, etc. (14). Yellow rice wine enhances the dissolution of active compounds, boosting the medicine’s effectiveness. Therefore, it is often used as an auxiliary material in the processing of *Polygonatum*. Research has shown that steaming with yellow rice wine increases the production and accumulation of volatile compounds, such as 5-hydroxymethylfurfural, in herbs like *Polygonatum*, *Cornus officinalis*, and *Schisandra chinensis* (15). Traditional Chinese medicine theory posits that honey, black beans, and cooked *Rehmannia glutinosa* can serve as excipients that enhance the effects of medicinal materials when their properties align with the “guijing” of the herbs. For instance, honey is believed to strengthen the spleen and lungs, while cooked *Rehmannia glutinosa* nourishes blood and yin. Steaming with black beans not only nourishes the kidneys and essence but also detoxifies. Specifically, when black beans are processed with *Aconitum carmichaelii*, *Aconitum kusnezoffii*, or *Curculigo orchioides*, they can promote the degradation and transformation of toxic alkaloids, thereby mitigating toxicity. Additionally, co-preparation with *Polygoni Multiflori* can reduce the concentration of bound anthraquinone (a component associated with diarrhea) and alleviate its purgative effect (16). Both black beans and *Polygonatum* share similar properties and are associated with the spleen and kidney meridians. Therefore, steaming them together can enhance *Polygonatum*’s effects in nourishing the kidneys and replenishing essence. Modern research has indicated anthocyanins and isoflavonoids—such as daidzein and daidzin—as the primary active ingredients in black beans, which promote growth and development, improve immunity, and support bone health. These pharmacological effects align with the kidney-tonifying properties of *Polygoni Multiflori*, *Polygonatum* (16).

The 2020 edition of the *Chinese Pharmacopoeia* lists yellow rice wine as the primary excipient for processing. *Polygonatum* steamed with black beans is mainly included in the processing specifications of Yunnan, Sichuan and other places. Current research on *Polygonatum* processing has primarily focused on *P. sibiricum* and *P. cyrtonema*, with limited studies on PK. Meanwhile, investigations into auxiliary materials have emphasized yellow rice wine, while the synergistic role of black beans—particularly their influence on the generation and accumulation of active components—remains poorly understood. Additionally, most previous studies have analyzed individual components (e.g., polysaccharides, saponins, or flavonoids) rather than offering a comprehensive view of dynamic metabolic changes across the entire processing cycle. These limitations impede the optimization of traditional methods and the scientific validation of their efficacy.

To address these gaps, we hypothesize that steaming PK with black beans promotes the production and accumulation of specific bioactive compounds, thereby enhancing its nutritional and health-promoting qualities. Our research aims to characterize the quality changes of black bean-processed PK through nine iterative steaming cycles and explore the influence of black beans on the active constituents of PK. By integrating quantitative analysis of key quality components, color measurement, sensory evaluation, and metabolomic analysis, this study provides the first comprehensive evaluation of black bean-steamed PK. The findings offer a scientific basis for optimizing traditional processing techniques and advancing the development of functional foods and herbal medicinal products.

## 2 Materials and methods

### 2.1 Preparation of PK with nine steaming cycles

The rhizomes of PK were collected from Yongping County, Dali, Yunnan Province, China (99.54°E, 25.46°N) and identified by Dr. Pengzhang Ji, Yunnan University of Chinese Medicine. To prepare the raw PK (S), fibrous roots were removed, and the fresh rhizomes were washed, sliced, then dried at 60°C in an electric blast drying oven (DHG-9140A, Taihongjun Scientific Instrument Co., Ltd., Guangdong, China). Of the remaining rhizomes, larger pieces were cut in half. Following a PK-to-black bean ratio of 4:1 (17), black beans were added, and the PK was steamed at atmospheric pressure in a 304 thickened stainless steel steam pot for 8 h, during which the steam condensate was collected throughout the process. After each steaming cycle, one group was removed, cooled, sliced uniformly, soaked in the collected steam condensate for 1.5 h, and dried at 60°C. Remaining PK was also dried at 60°C after each steaming, once it was ~80% dry, the PK was subjected to the same steaming and drying process, repeated for a total of nine cycles. This yielded nine groups of samples (P1–P9) with varying steaming intensities. The completely dried samples were then crushed and passed through a 100-mesh sieve for uniformity.

### 2.2 Sensory evaluation

Based on the literature’s description of steamed *Polygonatum*—“as black as paint and as sweet as syrupy sugar”—and adopting the method of Zheng et al. (18), the samples were assessed using the following criteria: “rich aroma, dark color, sufficient sweetness without numbing the tongue or having an odor, soft, moist, and sufficiently sticky texture.” Eight experienced testers would evaluate 10 groups of PK samples for aroma, color, taste, and texture. They will assign corresponding evaluation terms and scores, and a weighted scoring method will be used to calculate the total score. Table 1 shows the total scores and weights of the indicators.

**Table 1:** Total scores and weights of sensory evaluation indicators.

Indicator	Aroma	Color	Taste	Texture
Total score	100	100	100	100
Weight	10%	20%	40%	30%

### 2.3 Color measurement

The color of the resulting fine powder was measured with an NR110 colorimeter (3nh Technology, Shenzhen, Guangdong, China) and quantified using the LAB color system recommended by the International Commission on Illumination (CIE). The L*, a*, and b* values were recorded based on comparisons with standard white and blackboards. The total chromaticity, represented as E*ab, was calculated according to the following formula: E*ab = (L*^2^ + a*^2^ + b*^2^)^1/2^.

### 2.4 Determination of main quality components of steamed PK

The contents of polysaccharides and reducing sugars were measured using D-anhydrous glucose as the standard, with the phenol-concentrated sulfuric acid method and the DNS method employed, respectively. These measurements were conducted at wavelengths of 490 nm and 540 nm using a UV spectrophotometer (19, 20). The saponin content was determined using the vanillin-glacial acetic acid method, with a UV–visible spectrophotometer set to a wavelength of 550 nm and ginsenoside Rb1 serving as the standard (21). Total flavonoids were quantified using the sodium nitrite-aluminum nitrate method, with absorbance measured at 510 nm and rutin as the standard (22).

### 2.5 Untargeted metabolomic analysis

#### 2.5.1 Sample extraction and preparation

A sample weighing 60 mg was placed into a 1.5 mL centrifuge tube. Two small steel beads and 600 μL of a methanol–water solution (V: V = 7:3, including a mixed internal standard of 4 μg/mL) were added, and the mixture was pre-cooled in a −40°C refrigerator. After 2 min of cooling, the tube was placed into a grinder and ground at 60 Hz for 2 min. Following grinding, ultrasonic extraction was performed in an ice-water bath for 30 min. The sample was then allowed to stand at −40°Covernight and centrifuged at low temperatures for 10 min at 12,000 rpm and 4°C. A syringe was used to Aspirate 150 μL of the supernatant, which was then filtered using a 0.22 μm organic phase pinhole filter. The filtered sample was transferred to an LC injection vial and stored at −80°C until LC–MS analysis. Quality control samples (QC) were prepared by mixing equal volumes of extracts from all samples. All extraction reagents were pre-cooled to −20°C before use.

#### 2.5.2 LC–MS/MS untargeted metabolite detection

A Waters ACQUITY UPLC I-Class Plus/Thermo QE system (Waters Corporation/Thermo Fisher Scientific, MA, United States) equipped with an ACQUITY UPLC HSS T3 column (100 mm × 2.1 mm, 1.8 μm; Waters Corporation, Milford, MA, USA) was utilized for the isolation and detection of metabolites. The UPLC analysis parameters were set as follows: column temperature at 45°C; mobile phase A as water containing 0.1% formic acid and mobile phase B as acetonitrile; flow rate at 0.35 mL/min; injection volume at 3 μL. The elution gradient was programmed as follows: 0–2 min, 5% B; 2–4 min, 5–30% B; 4–8 min, 30–50% B; 8–10 min, 50–80% B; 10–14 min, 80–100% B; 14–15 min, 100% B; 15–15.1 min, 100–5% B; 15.1–16 min, 5% B. The mass spectrometry conditions included a spray voltage of -3000 V/3800 V in negative/positive ion mode, a capillary temperature of 320°C, an auxiliary gas heater temperature of 350°C, sheath gas flow rate at 35 Arb, auxiliary gas flow rate at 8 Arb, S-Lens RF level at 50, mass range from 70–1,050 m/z, full MS resolution at 70,000, MS/MS resolution at 17,500, and NCE/stepped NCE values at 10, 20, and 40.

#### 2.5.3 Qualitative and quantitative data

Metabolomic data were processed using Progenesis QI v3.0 software (Nonlinear Dynamics, Newcastle, United Kingdom) for baseline filtering, peak identification, integration, retention time correction, peak alignment, and normalization. Metabolites were identified and analyzed by referencing accurate mass and charge based on retention time (RT) against the Human Metabolome Database (HMDB), LipidMaps (v2.3), METLIN, and the Lu-Met-Plant3.0 local databases (Lu Ming Biotech Co., Ltd., Shanghai, China). Identification was based on m/z ratio, secondary fragmentation, and isotope distribution.

### 2.6 Statistical analysis

One-way analysis of variance (ANOVA) was conducted using IBM SPSS Statistics 27.0 (Armonk, NY, United States), with Tukey’s honestly significant difference (HSD) test determining significant differences. OriginPro 2022 software (Hampton, MA, United States) was used for principal component analysis (PCA), hierarchical cluster analysis (HCA), and bar graph creation. OECloud^1^ was used to generate heat maps and bar graphs for up-and down-regulated differential metabolites.

## 3 Results

### 3.1 Sensory and chromatic changes during the whole steaming process of PK

**Figure 1:**
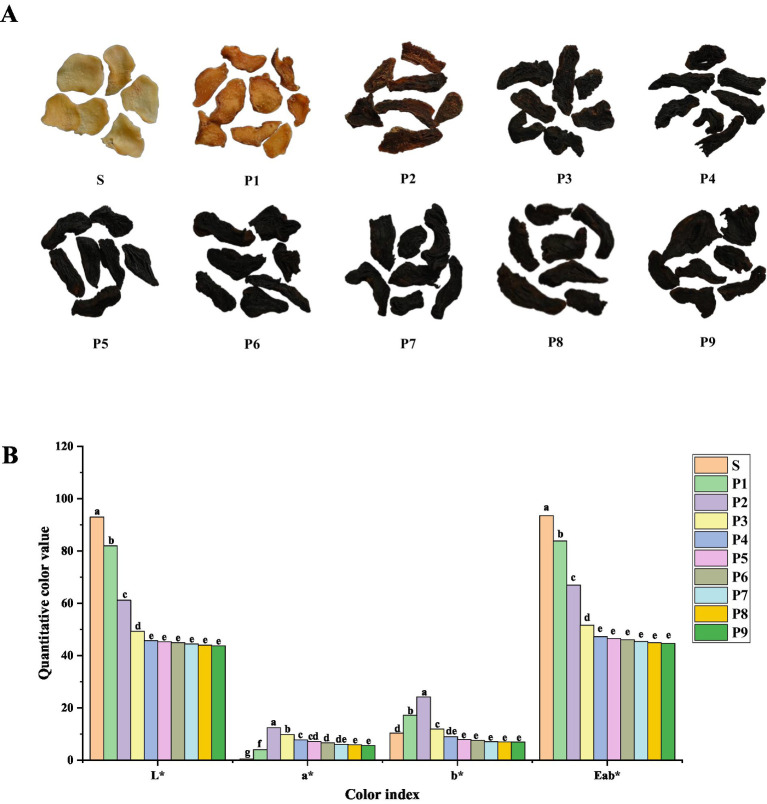
**(A)** Photographs and **(B)** color measurements of PK under different steaming cycles (different lowercase letters indicate significance *p* < 0.05).

With each successive steaming, the color of PK deepens gradually from beige to black (Figure 1A; Table 2) and stabilizes after four steamings. The quantitative color measurements (Figure 1B; Supplementary Table S1) indicate significant changes (*p* < 0.01) in each chromaticity index throughout the S–P4 stages. Over the entire processing period, brightness (L*) and total chroma (Eab*) decrease consistently, while the a* (red-green) and b* (yellow-blue) values first increase and then decline, with noticeable shift observed after P2. This trend aligns with the transformation of PK from yellow to brown and finally to a black or black-brown color, closely matching the sensory description results. Furthermore, after three rounds of steaming, the numbing effect of PK on the tongue disappeared. Its taste turned sweet, and its texture became soft and moist, earning an overall score of 94. However, additional steaming rounds brought out bitterness, sourness, astringency, and a burnt flavor. Therefore, this study concludes that steaming PK for about three rounds results in a better taste.

**Table 2:** Sensory description of PK under different steaming cycles.

Sample	Aroma	Color	Taste	Texture	Score
S	Almost no aroma	Beige	Indifferent in taste, strong numbing sensation in tongue	Harder, almost non-viscosity, tough	55
P1	Fresh glutinous fragrance	Brownish yellow	Slightly sweet, with heavy numbness	Harder, weakly viscous, tough	72
P2	Glutinous fragrance with caramel notes	Dark brown	Sweeter, slight numbness	Softer, viscous, slightly tough	81
P3	Caramel aroma	Brownish black	Sweet, no numbness	Soft and moisturizing, sufficient viscosity	94
P4	Caramel aroma	Jet black	Sweet, slightly bitter, no numbness	Soft and moisturizing, sufficient viscosity	91
P5	Burnt flavor	Jet black	Sweet, slightly bitter, no numbness	Soft and moisturizing, sufficient viscosity	89
P6	Burnt flavor	Jet black	Bitter, slightly sweet, sour, astringent	Soft and moisturizing, sufficient viscosity	87
P7	Burnt flavor	Jet black	Bitter, slightly acidic, almost no sweetness, heavy astringency	Soft, viscous	81
P8	Burnt flavor	Black-brown	Unsweet, slightly sour, astringent and bitter	Soft, viscous	73
P9	Burnt flavor	Black-brown	Unsweet, slightly sour, astringent and bitter	Soft, viscous	68

### 3.2 Changes in main quality components during the whole steaming process of PK

**Figure 2:**
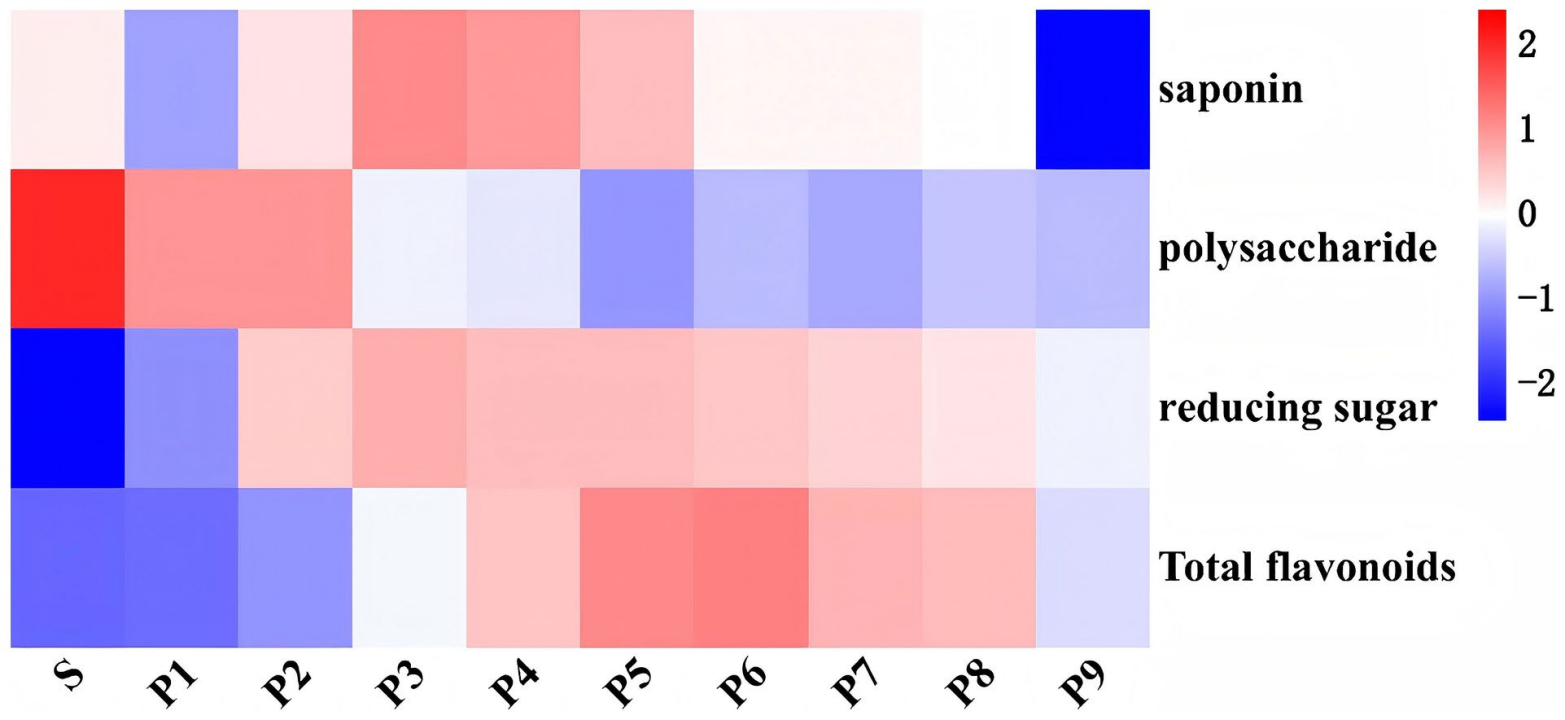
Thermogram of the main quality components of PK under different steaming cycles.

The results of the one-way analysis of variance and heat map analysis (Supplementary Table S2 and Figure 2) indicated that the contents of saponins, polysaccharides, reducing sugars, and flavonoids changed significantly during the steaming process (*p* < 0.05). Among these, the saponin content exhibited a wavy pattern, with the highest and lowest contents observed in P3 and P9, respectively. A highly significant increase in the total flavonoid content of PK was noted through steaming (*p* < 0.01). Among them, the total flavonoid content gradually accumulated from P1 to P6. However, after P6, a downward trend was observed, likely due to the degradation and transformation of flavonoids resulting from over-steaming (23). The polysaccharide content was highest in raw PK and decreased significantly after steaming (*p* < 0.05). This decrease occurred almost gradually with the increasing steaming times, consistent with the findings of Su et al. (24). In contrast, the reducing sugar content increased significantly after steaming (*p* < 0.01), reaching its peak at P3 before declining. This change in sugar content can be attributed primarily to the decomposition of polysaccharides and the accumulation of monosaccharides during the steaming process (25), while the decrease in reducing sugar content during the later stages of steaming may be associated with their consumption in the Maillard reaction and structural degradation caused by excessive steaming. Nonetheless, the trend in reducing sugar content observed in this study was both similar to and distinct from the results reported by Wang (25) and Wu et al. (26). Factors such as differences in basic conditions, including raw material varieties and steaming times, contributed to these discrepancies. Additionally, 25% black beans were incorporated into the PK processing and steamed together in this experiment.

### 3.3 Overall metabolomic analysis of PK processing process

**Figure 3:**
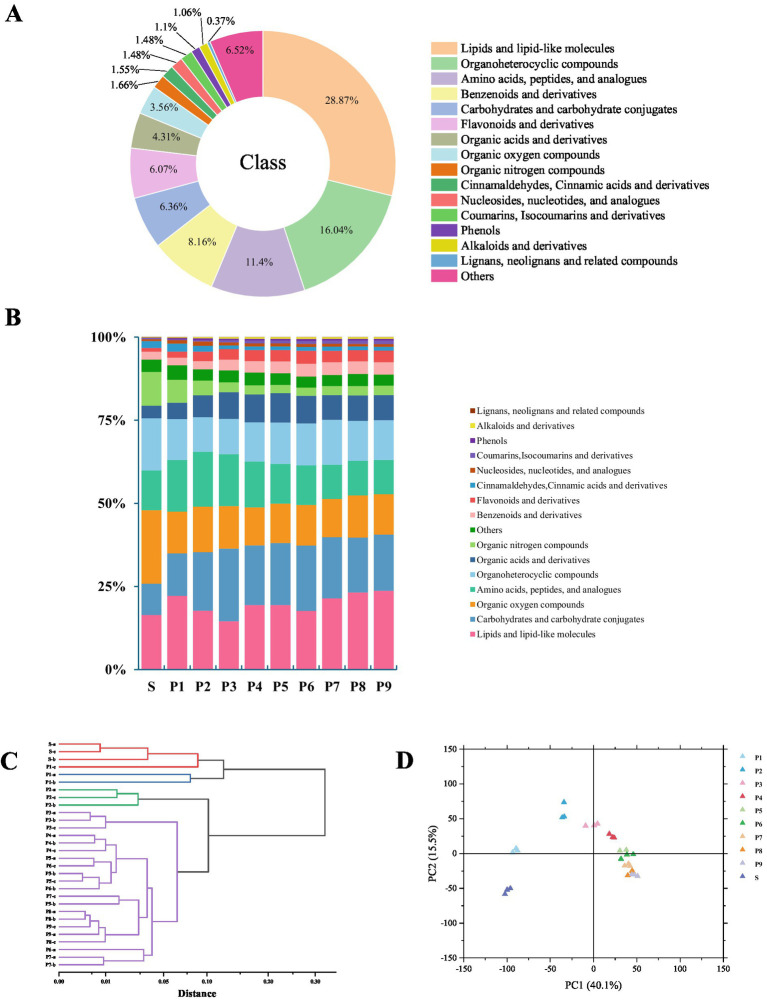
Results of metabolite analyses of PK: **(A)** Metabolite classification chart, **(B)** grouped percentage stacked bar plot of total metabolite expression abundance, **(C)** hierarchical cluster analysis (HCA) plot, and **(D)** principal component analysis (PCA) plot.

To further understand the influence of steaming cycle on the types and relative levels of PK metabolites, a non-targeted metabolomic analysis was performed on raw PK rhizome (S) and nine groups of steamed rhizomes (P1–P9). A total of 7,281 metabolites across 16 categories were identified (Figure 3A). This included 2,102 lipids and lipid-like molecules, 1,168 organoheterocyclic compounds, 830 amino acids, peptides, and analogs, 594 benzenoids and derivatives, 463 carbohydrates and carbohydrate conjugates, 442 flavonoids and derivatives, 314 organic acids and derivatives, 259 organic oxygen compounds, 121 organic nitrogen compounds, 113 cinnamaldehyde, cinnamic acid, and derivatives, 108 nucleosides, nucleotides, and analogs, 108 coumarins, isocoumarins, and derivatives, 80 phenols, 77 alkaloids and derivatives, 27 lignans, neolignans, and related compounds, and 475 other compounds. Compared with the raw rhizome (Figure 3B), steaming increased the proportion of carbohydrates, amino acids, organic acids, flavonoids, coumarins, phenols, alkaloids, and other compounds in PK.

The PCA model diagram obtained through 7-fold cross-validation (Supplementary Figure S1) demonstrates that the quality control samples (QC) are closely clustered, indicating good stability and repeatability in the experiment. Hierarchical cluster analysis (HCA) results (Figure 3C) reveal that PK with different steaming cycles can be categorized into four groups: raw rhizome (S), 1-time steaming (P1), 2-time steaming (P2), and 3–9 times of steaming (P3–P9). This clustering suggests that significant changes in metabolites occurred during the transition from S to P3 (*p* < 0.05), with stabilization of these changes observed after P3. The planar PCA results (Figure 3D) indicate that the variance contribution rate of principal component 2 across the 10 groups of samples with varying steaming cycles initially increased and then decreased. This trend suggests that some characteristic metabolites may be lost in the later stages of steaming. These metabolites primarily include amino acids, vitamins, lipids, carbohydrates, and related compounds, which are crucial for enhancing product flavor/texture, providing energy for the body, and promoting metabolic processes.

### 3.4 Comparative analysis of differential metabolites of PK at different processing levels

**Figure 4:**
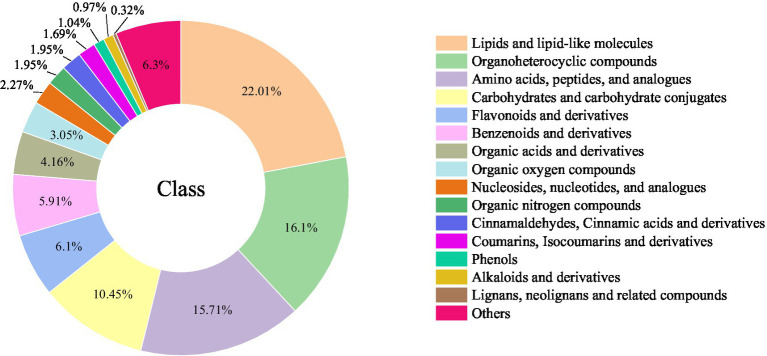
Differential metabolite classification chart.

In total, 1,540 differential metabolites (VIP > 1.0, *p* < 0.05) were identified from PK samples with different steaming cycles. Among these (Figure 4), there were 339 lipids and lipid-like molecules, 248 organoheterocyclic compounds, 242 amino acids, peptides, and analogs, 161 carbohydrates and carbohydrate conjugates, 94 flavonoids and derivatives, 91 benzenoids and derivatives, 64 organic acids and derivatives, 47 organic oxygen compounds, 35 nucleosides, nucleotides, and analogs, 30 cinnamaldehydes, cinnamic acid and derivatives, 30 organic nitrogen compounds, 26 coumarins, isocoumarins and derivatives, 16 phenols, 15 alkaloids and derivatives, 5 lignans, neolignans, and related compounds, and 97 others.

Supplementary Table S3 and Supplementary Figure S2 present quantitative information on the up-and down-regulated metabolites among the 10 sample groups (VIP > 1.0, *p* < 0.05, FC > 1.0 or FC < 1.0). Comparison revealed a significant increase (*p* < 0.05) in the number of metabolites regulated during the early stage of processing, particularly between S–P1 and P1–P2. The S–P1 stage regulated 279 metabolites, comprising 158 up-regulated and 121 down-regulated species, while the P1–P2 stage regulated 486 metabolites, including 278 up-regulated and 208 down-regulated species. The P2–P3 stage regulated 485 differential metabolites, with the highest number of up-regulated metabolites (357). Following four steamings, a decrease in the number of regulated metabolites was observed, leading to a gradual stabilization.

**Figure 5:**
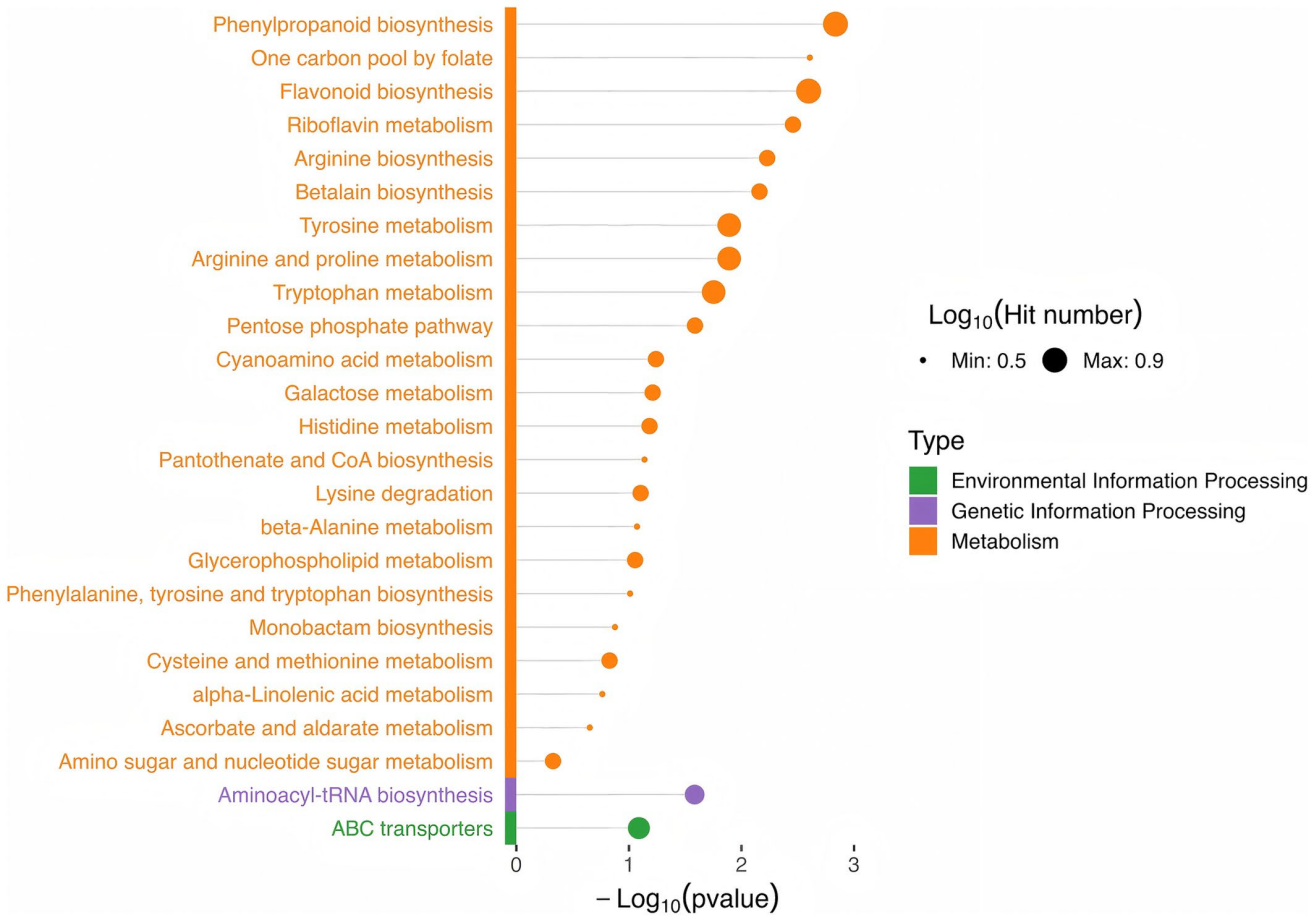
Top 25 pathways of differential metabolite enrichment.

KEGG pathway enrichment analysis was conducted on the screened differential metabolites (Supplementary Table S4; Figure 5). The results revealed significant enrichment in pathways related to amino acid metabolism and biosynthesis, such as tyrosine, tryptophan, arginine and proline, histidine, cysteine and methionine metabolism, as well as cyanoamino acid metabolism, betalain biosynthesis, arginine biosynthesis, and lysine degradation. Vitamin metabolic pathways were also enriched, including ascorbate and aldarate metabolism, pantothenate and CoA biosynthesis, riboflavin metabolism, and one-carbon pool by folate. Additionally, pathways for carbohydrate transformation and metabolism, such as the pentose phosphate pathway, galactose metabolism, and amino sugar and nucleotide sugar metabolism, were enriched. Other enriched pathways included flavonoid biosynthesis, alpha-linolenic acid metabolism, monobactam biosynthesis, glycerophospholipid metabolism, and phenylpropanoid biosynthesis.

### 3.5 Dynamic analysis of main differential metabolites in the whole process of processing PK

**Figure 6:**
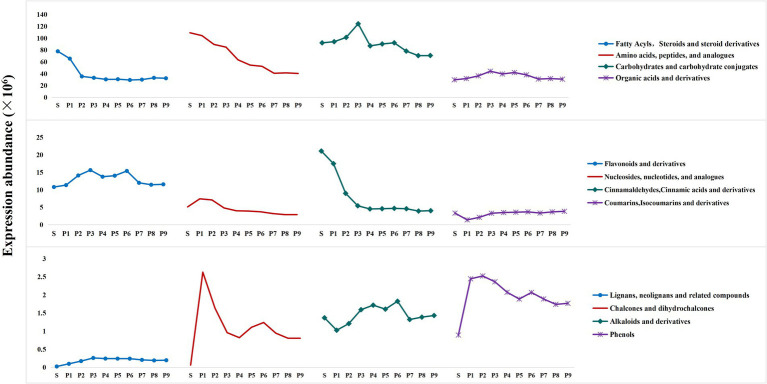
Overall trends in the concoction process of several major differential metabolites.

To assess the dynamic changes of differential metabolites throughout the PK process, an overall single-factor analysis of variance (ANOVA) (Supplementary Table S5, Figure 6) and cluster heatmap analysis (Supplementary Figure S3) were performed on major categories of differential metabolites. Their concentrations are expressed as mass spectrum abundance values.

#### 3.5.1 Fatty acyls, steroids, and derivatives

As shown in Supplementary Figure S3A, among the 144 fatty acyls and 38 steroids and derivatives analyzed, 37 metabolites were significantly reduced after steaming (*p* < 0.05). In contrast, the abundance of 80 metabolites either stabilized or increased progressively with the degree of steaming, suggesting that these metabolites undergo biotransformation during this process. The abundance of 66 metabolites was relatively high in stages P1 through P4, although prolonged steaming led to a decline. A one-way ANOVA indicated a significant decrease in the levels of fatty acyls, steroids, and their derivatives during steaming (*p* < 0.05), with a trend toward stabilization after P2. Besides the biotransformation of metabolites, prolonged and repeated high-temperature exposure led to the loss of certain compounds and structural degradation.

#### 3.5.2 Amino acids, peptides, and analogs

Amino acids are important nutrients and flavor components (27). It has been found that *Polygonatum* is high in amino acid content and rich in types (28). One-way ANOVA indicated that the expression abundance of amino acids, peptides, and their analogs was highest in raw PK, with an overall decreasing trend observed during the steaming process. The most significant decrease occurred after P4 (*p* < 0.05). The heat map analysis of 242 metabolites is shown in Supplementary Figure S3B. The amino acids detected in this study, including L-Tyrosine, L-Arginine, L-Aspartic Acid, L-Histidine, D-Lysine, and D-Ornithine, exhibited a downward trend after steaming. This trend aligns with the changes observed in amino acids in PK made from yellow rice wine (29). Most corresponding aldehydes, acids, alcohols, esters, and other derivatives increased after steaming. This increase is primarily attributed to the decarboxylation, transamination and dehydrogenation reactions of amino acids. Furthermore, participation in the synthesis of aromatic compounds and pigments may contribute to the continuous reduction of amino acid metabolites during the steaming process (30).

#### 3.5.3 Carbohydrates and carbohydrate conjugates

A total of 161 differential metabolites were identified from the detected carbohydrates and their conjugates (Supplementary Figure S3C). Polysaccharides (Amylopectin, Inulobiose, etc.) and certain oligosaccharides (including cellotetraose, 3-Beta-Glucosylcellotriose, Cellopentaose, etc.) are gradually hydrolyzed into smaller molecular weight oligosaccharides and simple sugar through the breakage of glycosidic bonds during repeated heating and cooking. This process promotes the accumulation of small molecule sugars, which exhibit higher expression abundance in P1–P3. D-Fructose, D-Apiose, Beta-D-Galactose, 2,5-Anhydro-D-Mannose, D-Altro-D-Manno-Heptose, and Kinetin-9 -N-Glucoside, Difructose Anhydride III, 9-O-Acetylneuramine Acid, etc. reach their highest levels in P3 and are the main differential metabolites influencing the overall trend of sugar changes. In the later stages of steaming, small molecule sugars are reduced, possibly due to their combination with flavonoids, coumarins, terpenes, and other compounds to form corresponding glycosides. One-way analysis of variance results for total carbohydrates indicated that the expression abundance of total sugars increased during the S-P3 process, peaked at P3, and then decreased significantly (*p* < 0.05). The changes in sugar content during the steaming process are consistent with the detection results of the main quality components.

#### 3.5.4 Organic acids, nucleosides and nucleotides, cinnamic acid, and corresponding derivatives

Among the 64 organic acids and their derivatives (Supplementary Figure S3D), the degradation and transformation of 14 organic acids and their derivatives, including Gamma-Delta-Dioxovaleric Acid, Phosphoglycolic Acid, 3-Hydroxysebacic Acid, and 4-Carboxy-2-Hydroxy-Cis, Cis-Muconic Acid, were promoted by heat treatment. This treatment resulted in a significant decrease in their expression abundance after steaming (*p* < 0.05). In contrast, the expression of 12 organic acids and derivatives, such as Gallicynoic Acid I, 3-Oxoglutaric Acid, 3-Oxo-5 s-Amino-Hexanoic Acid, and 6-Guanidino-2-Oxocaproic Acid, significantly increased during phases P1 to P3 (*p* < 0.05) before experiencing a substantial decrease. *D*-Malic Acid and (S)-3-Sulfonatolactate exhibited fluctuating changes during the steaming process. Additionally, the remaining 16 carboxylic acids and derivatives, along with 8 keto acids, 6 hydroxy acids, 4 phosphoric acids and their derivatives, and two types of hybrid peptides gradually accumulated and stabilized during repeated steaming and drying. One-way ANOVA revealed an overall trend of increasing and then decreasing expression levels of PK organic acids and derivatives throughout the steaming process, with the lowest abundance observed in S and the highest in P3.

Studies have shown that the presence of nucleotides enhances taste and palatability (27). The heat map analysis results of 35 nucleosides, nucleotides, and analogs are presented in Supplementary Figure S3F. The expression levels of 9 nucleosides, 4 nucleotides, and 5 analogs increased or fluctuated slightly with an increase in steaming times. Additionally, 15 types of nucleosides and analogs, including 3-Deoxyguanosine, Adenosine, Guanosine, and 3´-Amino-3´-Deoxythimidine, exhibited higher expression levels in S or P1-P3 stages. Notably, the P1 and P2 stages promoted the significant accumulation (*p* < 0.05) of 13 nucleosides and analogs, including 3-Deoxyguanosine, 5´-Methylthioadenosine, 5-Hydroxymethyl-2´-Deoxyuridine, and N6-Phenylisopropyladenosine, contributing to the overall expression levels of nucleosides, nucleotides, and analogs. After multiple steaming processes, these metabolites gradually decreased due to transformation or loss. The overall one-way ANOVA results indicated that the expression levels of nucleosides, nucleotides, and analogs initially increased and then continued to decrease during the steaming process, with the highest expression levels observed in the P1 and P2 stages.

The overall one-way analysis of variance results indicated that the total expression abundance of cinnamaldehydes, cinnamic acids, and their derivatives was highest in S. This abundance decreased with increasing steaming cycles and ultimately stabilized. However, among the 30 analyzed cinnamaldehydes, cinnamic acids and derivatives (Supplementary Figure S3G), 22 exhibited increases after steaming, with many either increasing or changing slightly as steaming progressed. This observation is inconsistent with the overall trend in expression abundance. Notably, the changes observed in (E)-3-(2-Hydroxyphenyl)-2-Propenal, 2-Hydroxycinnamic Acid, and Trans-Cinnamic Acid throughout the steaming process closely aligned with the overall changes in expression abundance. The expression levels of these three metabolites in S were exceptionally high, contributing 97.47% to the total abundance. It can be speculated that (E)-3-(2-Hydroxyphenyl)-2-Propenal, 2-Hydroxycinnamic Acid, and Trans-Cinnamic Acid are the primary metabolites of this compound class in the raw PK of this study and may serve as characteristic compounds for assessing changes in cinnamaldehydes, cinnamic acids, and their derivatives during steaming.

#### 3.5.5 Flavonoids, alkaloids, phenols, and corresponding derivatives

The differential metabolite heat map analysis results for the 94 screened flavonoids and their derivatives are shown in Supplementary Figure S3E. These metabolites are classified into six subcategories: 68 flavonoids, 18 isoflavonoids, 4 neoflavonoids, 2 aurone flavonoids, 1 homoisoflavonoid, and 1 2-arylbenzofuran flavonoid. During the steaming process, the abundance of only 14 metabolites showed a downward trend. These include Epicatechin-(4β → 8)-Gallocatechin, Puerarone, and 12 flavonoid glycosides such as Primflaside, Cyanidin 3-Arabinoside, Neobignonoside, Isomollupentin 4′-O-Glucoside, Quercetin 3-(2″-Caffeoylsambubioside)-7-Glucoside, and Apigenin 7-(6″-Malonylneohesperidoside). The remaining metabolites increased in abundance after steaming. Among these, 15 metabolites—including Naringenin, Cajanol, Mosloflavanone, Flavonol 3-O-D-Glucoside, and 5,7-Dihydroxy-4′-Methoxy-8-Methylflavanone—were highly expressed in P1 and P2, suggesting that controlled steaming promotes their accumulation, whereas repeated steaming leads to a reduction in their levels. Conversely, 7 catechins ((+)-Catechin, Catechin 7-Arabinofuranoside, Catechin Tetramethylether, Epicatechin 3′,4’-Dimethyl Ether, Epicatechin Pentaacetate, 4′-Methyl-(−)-Epigallocatechin 3-(4-Methyl-Gallate), and 4′-O-Methylepicatechin 7-O-Glucuronide), 35 flavonoids (including Sakuranetin, Myricetin 3,3′,4′-Trimethyl Ether, Leucopelargonidin 3-O-Glucoside, Apigenin 7-(6″-Ethylglucuronide), and 7,4′-Dihydroxyflavone), 13 isoflavones (including Daidzein and Daidzin, the main active components in black beans, along with Glycyrol and Parvisoflavone), 3 neoflavonoids (Dalbergin, Nordalbergin, and 7,3′-Dihydroxy-5,4′-Dimethoxy-6-Formyl-4-Phenylcoumarin), 2 aurone flavonoids (4′,6-Dihydroxy-3′-Methoxyaurone and Amaronol B), and 1 2-arylbenzofuran flavonoid (2′,4′-Dihydroxy-5,6-Methylenedioxy-2-Phenylbenzofuran) maintained high abundance levels after multiple steamings, stabilizing or reaching peak expression by P3.

The overall results of the one-way analysis of variance regarding flavonoids and their derivatives indicated that the expression abundance of total flavonoids first increased during the steaming process and then decreased, almost reaching the level of S after P7. Notably, high expression abundance was observed in P3 and P6, with P3 showing the highest level. Previous studies have demonstrated that the total flavonoid content in *Polygonatum* continues to rise with an increasing number of steaming cycles (13, 29), primarily due to differences in auxiliary materials and steaming duration. Furthermore, some flavonoids may lack functional groups that react with color- rendering agents (e.g., 3 or 5-hydroxyl, o-diphenol hydroxyl), which prevents their detection by UV spectrophotometer. This limitation may lead to discrepancies between the results of the main mass component assay and those of the differential metabolite analyses. To overcome this limitation, the liquid chromatography-mass spectrometry (LC–MS) method can be employed to enhance the precision of detection.

The one-way analysis of variance revealed that alkaloids and their derivatives exhibited an overall wavy pattern during the steaming process, with the highest expression abundance occurring at P6. Steaming treatment resulted in a significant increase in phenolic components (*p* < 0.05), their expression abundance peaked between P1 and P3 before demonstrating a downward trend following multiple steaming treatments. The heat map analysis of alkaloids and derivatives, and phenols is presented in Supplementary Figure S3H. Among the findings, Emetine and Trigonelline showed significant reductions after steaming (*p* < 0.05), while Prosopinine and Galanthaminone displayed significant increases at P1 and P2, respectively (*p* < 0.05), before gradually decreasing with continued steaming. Additionally, repeated heating treatments promoted the increase and stabilization of 11 alkaloids, such as Harmine, Morphine-6-Glucuronide, (−)-Cytisine, Pilocarpine, and (+)-Lysergic Acid. The expression abundance of 16 phenolic differential metabolites significantly increased after steaming (*p* < 0.05), with certain metabolites, such as Betaxolol, Cardanoldiene, Dopamine, and Rimiterol, exhibiting higher expression abundance during the first three steaming cycles.

#### 3.5.6 Coumarins, lignans, chalcones, and corresponding derivatives

The heat map analysis results for coumarins, lignans, chalcones, and their derivatives are displayed in Supplementary Figure S31. The steaming treatment significantly reduced the levels of three coumarins—3-Hydroxycoumarin, Clausarinol, and Pectachol—by promoting their hydrolysis and transformation (*p* < 0.05). Conversely, 23 other coumarins and coumarin glycosides, such as Byakangelicin, Dihydroxybergamottin, 8-Methoxycoumarin, Methoxsalen, and Scopoletin, along with five lignans and five chalcones, showed a marked increase following steaming (*p* < 0.05). Specifically, 4,2′,4′-Trihydroxy-6′-Methoxydihydrochalcone and Lanceoletin exhibited higher expression levels in P1 and P2, suggesting that short-term heating enhanced their concentration; however, repeated steaming likely led to degradation due to structural damage. In contrast, compounds such as 2 h-1-Benzopyran-2-One, Aminomethyl-, 4-Methyldaphnetin, 4-Methylumbelliferyl Beta-D-Xylopyranoside, Dihydroxybergamottin, and (8R,8′R,9S)-9-Hydroxy-3,4-Dimethoxy-3′,4′-Methylenoxy-9,9′-Epoxylignan showed higher abundance after prolonged deep steaming, especially Dihydroxybergamomottin, which accumulated significantly after P6 following a near-zero level during the S-P5 phases (*p* < 0.05). Overall, the one-way ANOVA results indicated that, except for significant decreases at P1 and P2 (*p* < 0.05), levels of coumarins, isocoumarins, and their derivatives increased to levels comparable with those in S by P3. The elevated abundance after steaming was mainly attributed to newly formed coumarins and glycosides, whereas the high levels in the S group were due primarily to the presence of 3-Hydroxycoumarin, Clausarinol, and Pectachol, designating these compounds as the primary coumarins in raw PK. Both lignans and chalcones increased significantly post-steaming (*p* < 0.05). Notably, lignans reached peak expression at P3 and then stabilized, while chalcones, due to contributions from 4,2′,4′-Trihydroxy-6′-Methoxydihydrochalcone and Lanceoletin, peaked at P1 and subsequently declined in a fluctuating pattern.

## 4 Discussion

**Figure 7:**
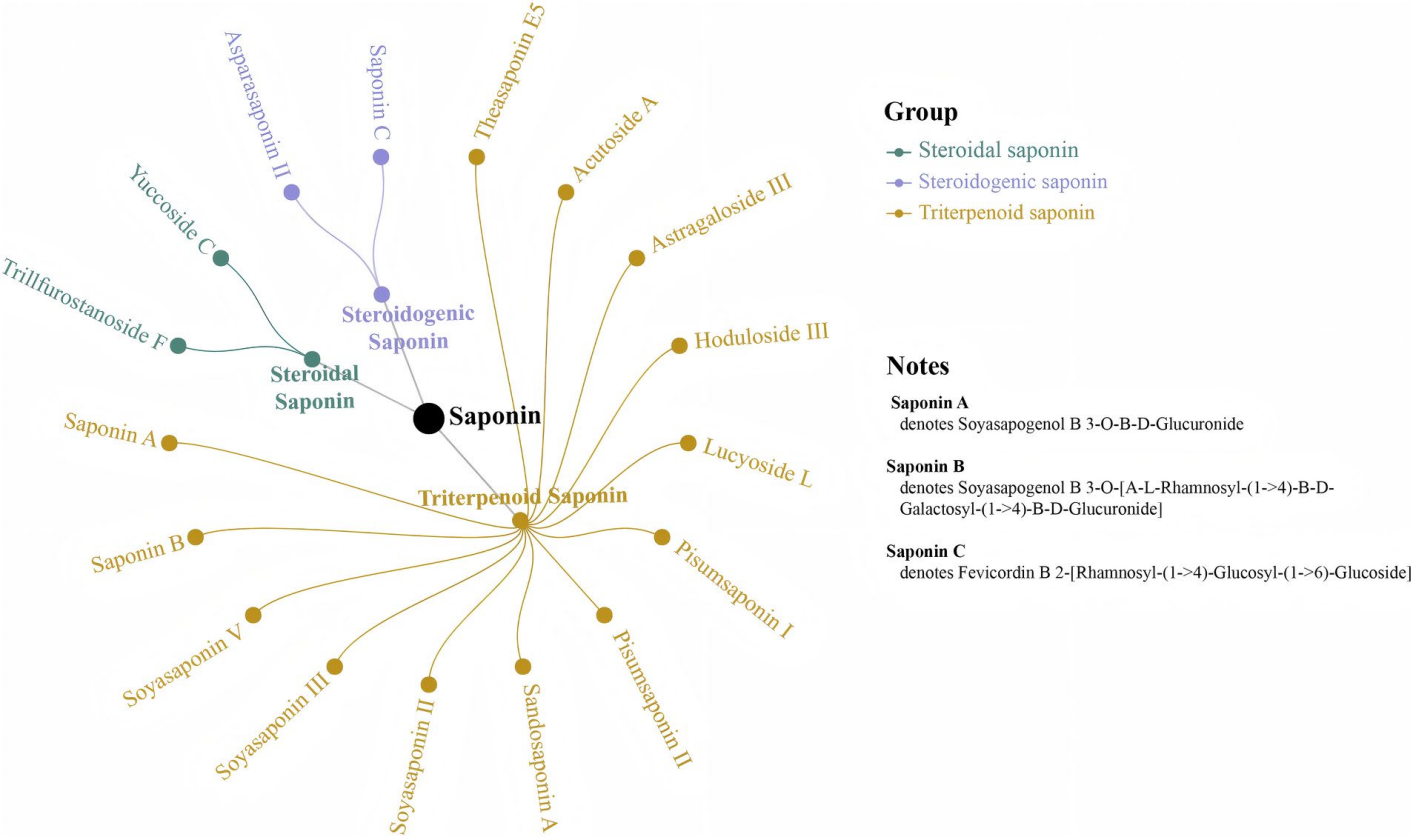
Seventeen newly produced saponins obtained post-steaming.

Flavonoids, known for their anti-aging, neurological, and vascular protective effects, as well as for enhancing physical fitness, are a vital part of the daily diet (31). Unlike yellow rice wine, black beans are especially rich in flavonoids (32). Following the steaming with black beans, the production and accumulation of five black bean-specific isoflavones Daidzin, Daidzein, 6″-O-Acetyldaidzin, Daidzein 4′-O-Glucuronide, and Daidzein 7-O-Glucoside-4′-O-Apioside—were observed in PK, with significant increases in Daidzin and Daidzein levels (*p* < 0.05). Additionally, the steaming process with black beans enhanced the accumulation of shared compounds in PK, including Naringenin, Naringin and (+)-Catechin. Among the differential metabolites identified, 25 saponin components (Supplementary Table S6) showed significant increases following steaming (*p* < 0.05), the majority of which were triterpene saponins. Of these, 17 saponins were newly produced after steaming, comprising 13 triterpene saponins, 2 steroid saponins, and 2 steroidogenic saponins (Figure 7). These saponins exhibit various physiological activities, including antioxidant, anti-inflammatory, hypoglycemic, and immune-regulating effects (33, 34). Previous studies have indicated that the saponins in *Polygonatum* are primarily steroidal saponins, with only more than 10 types of triterpene saponins identified (35, 36), which differ from those observed in this study. In contrast, the saponins present in black beans are predominantly pentacyclic triterpene saponins (37), including Sandosaponin A, Soyasaponin II, Soyasaponin III, Soyasaponin V, Pisumsaponin I, Pisumsaponin II, Soyasapogenol B 3-O-*β*-D-Glucuronide, and Soyasapogenol B 3-O-[*α*-L-Rhamnosyl-(1 → 4)-β-D-Galactosyl-(1 → 4)-β-D-Glucuronide], which were discovered in this study. Furthermore, the production and increase of new compounds, such as Theasaponin E5, Hoduloside III, and Lucyoside L, may result from the synergistic action of black beans and PK. Although the original saponins of PK, including Diosgenin, Ginsenoside, and Asiaticoside, were reduced during the steaming process, they were not significantly affected (*p* > 0.05).

Although the polysaccharide content of *Polygonatum* significantly decreased after steaming (*p* < 0.05), multiple studies have demonstrated that steamed *Polygonatum* exhibits higher pharmacological activity compared to raw *Polygonatum* (38–40), which is closely associated with the steaming method and sustained high-temperature processing. High-temperature steaming disrupts the external structure of *Polygonatum*, increasing its contact area with digestive fluids or extraction solvents, and promotes enzymatic and hydrolytic reactions within the rhizomes (41). While steaming with yellow rice wine facilitates the release and dissolution of active substances, as well as the hydrolysis, substitution, and modification of components such as polysaccharides and saponins, reducing their molecular weight and enhancing their bioavailability (35, 42). This explains why higher levels of saponins, flavonoids, and other components are detected in *Polygonatum* steamed with yellow rice wine, with their content continuously increasing during the steaming process (29, 42). In contrast to studies using yellow rice wine as an excipient, most active components in this study reached their peak enrichment after three steaming cycles. More importantly, we revealed for the first time that steaming PK with black beans promotes a dual metabolic enhancement: (1) the exogenous incorporation of isoflavones (e.g., Daidzin and Daidzein) and triterpenoid saponins (e.g., Sandosaponin A, Soyasaponin II, and Pisumsaponin I) from black beans into PK, and the incorporation of black beans potentially providing conditions for the synthesis of new compounds such as Theasaponin E5; (2) the endogenous promotion of the accumulation of shared components, such as flavonoids and sugars. Research on the material changes during the steaming process of *Polygonum multiflorum* also revealed that co-steaming with black beans facilitates the accumulation of total sugars and the enrichment of flavonoid components (such as genistein, genistin and daidzein) in *Polygonum multiflorum*, and it has been proved that steaming *Polygonum multiflorum* with black beans can achieve the effect of “enhancing efficacy and reducing toxicity,” the changing trend of its saccharides is the same as that in this study (43). These newly formed active components are closely associated with promoting growth and development, delaying aging, and improving osteoporosis. In summary, while steaming with yellow rice wine focuses on enhancing overall efficacy, steaming with black beans emphasizes the improvement of specific functions, offering more targeted and precise effects. This study provides a theoretical basis for the synergistic enhancement of *Polygonatum*’s kidney-tonifying and essence-replenishing effects through the addition of black beans.

Besides the decrease in sugar content, the reduction in free amino acids, and the increase and persistence of catechins, aldehydes, acids, esters, alcohols, and other amino acid derivatives, may contribute to the diminished sweetness and the pronounced bitterness and astringency of *Polygonatum* in the later stages of steaming (44, 45). The emergence of sour taste may be linked to the increased levels and persistence of certain organic acids (46). It is generally accepted that the deepening color of *Polygonatum* after steaming primarily results from the progression of the Maillard reaction and the accumulation of melanoidins. During steaming, polysaccharides are extensively decomposed into monosaccharides, which enhances the sweetness of the steamed *Polygonatum*. Additionally, the rise in reducing sugar content, along with the presence of amino acids and phenols (47), creates favorable conditions for the Maillard reaction. Furthermore, the increase in flavonoids during steaming contributes to the darker color of *Polygonatum* (13). Variations in *Polygonatum* varieties, steaming conditions, and the addition of auxiliary materials lead to differences in the sensory quality and color presentation of various steamed *Polygonatum* at each stage (18, 48). However, a common observation is that repeated steaming and drying promoted the Maillard reaction and the accumulation of flavonoids and simple sugars. This process has established the characteristics of *Polygonatum*, described as “black as paint and sweet as syrupy sugar.”

This study provides critical insights for optimizing the processing protocols and enhancing the product quality of *Polygonatum*, while establishing a foundation for developing *Polygonatum*-based products with targeted therapeutic efficacy, such as nutraceuticals or dietary supplements.

## 5 Conclusion

As the degree of steaming increases, the color of PK gradually deepens and becomes black, while its taste becomes soft, glutinous, and sweet. Steaming three times yields an improved flavor. The analysis of intrinsic components revealed that PK is rich in lipids, amino acids, sugars, flavonoids, and organic acids. P1-P3 significantly impacted metabolite levels (*p* < 0.05), most metabolites, including carbohydrates, saponins, flavonoids, and organic acids, reached their peak enrichment at P3, after which the changes in metabolites stabilized. These metabolites are closely associated with the multifaceted biological activities of PK, including anti-inflammatory, antioxidant, metabolic regulation, and hypoglycemic effects. Compared to *Polygonatum* steamed without excipients or with yellow rice wine, steaming with black beans significantly enhances (*p* < 0.05) the diversity of metabolites in PK, such as flavonoids and saponins, particularly with a notable increase in triterpenoid saponins. This plays a crucial role in improving the efficacy and quality of steamed PK. In conclusion, using black beans as excipients is cost-effective, it reduces irritation from *Polygonatum* while effectively incorporating its active ingredients into *Polygonatum* to synergistically enhance therapeutic effects.

Based on the findings of this study, subsequent pharmacological investigations employing both *in vivo* and *in vitro* approaches are recommended to validate the therapeutic and health-promoting effects of steamed PK with black beans, with a focus on elucidating its kidney-tonifying effects, antioxidant properties, and immunomodulatory functions, as well as exploring the mechanisms of action of its characteristic bioactive compounds, such as daidzein and daidzin. Additionally, future systematic exploration, comparison, and optimization of different steaming methods for *Polygonatum* are also needed to establish optimal steaming conditions, standardize processing technology, and improve the quality of steamed *Polygonatum*.

## Data Availability

The raw data supporting the conclusions of this article will be made available by the authors, without undue reservation.

## Footnotes

^1^ https://cloud.oebiotech.com

## References

[1] Xu XR Shan MM Chu CQ Bie SK Wang H Cai SB. Polysaccharides from *Polygonatum kingianum* Collett & Hemsl ameliorated fatigue by regulating NRF2/HO-1/NQO1 and AMPK/PGC-1α/TFAM signaling pathways, and gut microbiota. Int J Biol Macromol. (2024) 266:131440. doi: 10.1016/j.ijbiomac.2024.131440, PMID: 38593898

[2] Qin PY Xu YJ Zou XD Duan JH Qiu B Li XF. Effect and mechanisms of *Polygonatum kingianum* (polygonati rhizome) on wound healing in diabetic rats. J Ethnopharmacol. (2022) 298:115612. doi: 10.1016/j.jep.2022.115612, PMID: 35987409

[3] Wang Z Liu H Fu RZ Ou JM Wang B. Structural characterization and anti-inflammatory activity of a novel polysaccharide PKP2-1 from *Polygonatum kingianum*. Front Nutr. (2023) 10:1156798. doi: 10.3389/fnut.2023.1156798, PMID: 37051130 PMC10083337

[4] Yang JJ Zhao SL Zhang X Ma YG Jiang JG. *Polygonatum kingianum* saponins delay cellular senescence through SASP down-regulation and prolong the healthy lifespan of *Caenorhabditis elegans* by activating sir-2.1/autophagy. Ind Crop Prod. (2023) 201:116903. doi: 10.1016/j.indcrop.2023.116903

[5] Yang XX Wei JD Mu JK Liu X Dong CJ Zeng LX. Integrated metabolomic profiling for analysis of antilipidemic effects of *Polygonatum kingianum* extract on dyslipidemia in rats. World J Gastroentero. (2018) 24:5505–24. doi: 10.3748/wjg.v24.i48.5505, PMID: 30622379 PMC6319135

[6] Liu S Jia QJ Peng YQ Feng TH Hu ST Dong JE. Advances in mechanism research on Polygonatum in prevention and treatment of diabetes. Front Pharmacol. (2022) 13:758501. doi: 10.3389/fphar.2022.758501, PMID: 35211009 PMC8861320

[7] Ren HM Zhang JL Deng YL Ye XW Xia LT Liu MM. Analysis of chemical constitutions of *Polygonatum cyrtonema* dried rhizomes before and after processing with wine based on UPLC-Q-TOF-MS. Chin J Exp Tradit Med Form. (2021) 27:110–21. doi: 10.13422/j.cnki.syfjx.20202147

[8] Wu WJ Wang YL Yi P Su XF Mi Y Wu L. Various steaming durations alter digestion, absorption, and fermentation by human gut microbiota outcomes of *Polygonatum cyrtonema* Hua polysaccharides. Front Nutr. (2024) 11:1466781. doi: 10.3389/fnut.2024.1466781, PMID: 39364149 PMC11446882

[9] Jin J Lao J Zhou RR He W Qin Y Zhong C. Simultaneous identification and dynamic analysis of saccharides during steam processing of rhizomes of *Polygonatum cyrtonema* by HPLC–QTOF–MS/MS. Molecules. (2018) 23:2855. doi: 10.3390/molecules23112855, PMID: 30400172 PMC6278431

[10] Mei XY Xia JB Li WQ Wu YF Cheng H Chen SG. Glycan degradation in Polygonati Rhizoma: effects of traditional 'nine steaming and nine basking' on low molecular weight Fructans and polysaccharides. Food Chem X. (2025) 25:102131. doi: 10.1016/j.fochx.2024.102131, PMID: 39850053 PMC11754683

[11] Shen JJ Pu WT Song QY Ye BH Shi XX Chen YW. Traditional processing can enhance the medicinal effects of *Polygonatum cyrtonema* by inducing significant chemical changes in the functional components in its rhizomes. Pharmaceuticals. (2024) 17:1074. doi: 10.3390/ph17081074, PMID: 39204179 PMC11359098

[12] Zhu SQ Liu P Wu WX Li D Shang EX Guo S. Multi-constituents variation in medicinal crops processing: investigation of nine cycles of steam-sun drying as the processing method for the rhizome of *Polygonatum cyrtonema*. J Pharmaceut Biomed. (2022) 209:114497. doi: 10.1016/j.jpba.2021.114497, PMID: 34871951

[13] Nie XH Wang LY Wang SY Yu NX Lu YC Lyu WD. In vitro hypoglycemic and antioxidant activities of steamed *Polygonatum cyrtonema* Hua with various steaming degrees: relationship with homoisoflavonoids. Food Biosci. (2023) 53:102518. doi: 10.1016/j.fbio.2023.102518

[14] Qin YW Zhang LP Zhao Q Bao KD Jiang CX. Research progress on *Polygonatum cyrtonema* processed by nine times steaming and nine times shining. Chin Tradit Herb Drug. (2020) 51:5631–7. doi: 10.7501/j.issn.0253-2670.2020.21.029

[15] Ou ZM Li XJ Zhang BB Tong Y Ma ZS Liu DW. Research status and prospect of Chinese medicine wine processing. Chin Arch Tradit Chin Med. (2021) 39:28–32. doi: 10.13193/j.issn.1673-7717.2021.12.006

[16] Liu CY Kang WQ Li M Ou L Bai Y Dong TW. History and research progress of processing auxiliary black soya bea. Guiding J Tradit Chin Med Pharm. (2023) 29:61–6. doi: 10.13862/j.cn43-1446/r.2023.01.011

[17] Yunnan Provincial Drug Administration. Yunnan provincial processing specifications for Chinese medicinal decoction pieces (Yun YPBZ-0066-2005-2023). Kunming: Yunnan Science and Technology Press (2023).

[18] Zheng XQ Xu C Jin CS Liu JL Liu CF Li L. Research on relationship between processing degree and internal and external quality of *Polygonatum cyrtonema* processed by “nine-steaming and nine-suncuring” based on color change. Chin Tradit Herb Drug. (2022) 53:1719–29. doi: 10.7501/j.issn.0253-2670.2022.06.014

[19] Wang YX Tang ZW Peng T Chen HL Gao TY He PY. Optimization of phenol sulfuric acid method for the polysaccharide content of wine-steamed *Polygonatum cyrtonema* Hua. Sci Technol Food Ind. (2021) 42:308–16. doi: 10.13386/j.issn1002-0306.2021010069

[20] Pan DM Cai WS Liang YT Shen Y Shi ZM Yi YK. Comparison of determination methods of saccharides in *Polygonatum cyrtonema* and optimization of its wine-steaming technology. China Pharm. (2021) 32:2994–3000. doi: 10.6039/j.issn.1001-0408.2021.24.09

[21] Tian XJ Luo XW Yang XZ Zhang W. The effects of different processing methods on components in *Polygonatum Kingianum* coll.Et Hemsl. Chem Reagents. (2021) 43:790–4. doi: 10.13822/j.cnki.hxsj.2021007907

[22] Cai Y Zhou SS Shi MF Wang JY Feng Q Zhang QW. Effects of fertilization on growth and primary metabolite accumulation in *Polygonatum cyrtonema*. J Chin Med Mater. (2022) 45:2805–11. doi: 10.13863/j.issn1001-4454.2022.12.004

[23] Sun Y Zhou L Shan X Zhao TT Cui MR Hao WQ. Untargeted components and in vivo metabolites analyses of Polygonatum under different processing times. Lwt. (2023) 173:114334. doi: 10.1016/j.lwt.2022.114334

[24] Su LL Li XM Guo ZJ Xiao XY Chen P Zhang JB. Effects of different steaming times on the composition, structure and immune activity of Polygonatum polysaccharide. J Ethnopharmacol. (2023) 310:116351. doi: 10.1016/j.jep.2023.116351, PMID: 36914038

[25] Wang JN Lu Q Xue SJ Chen XC Zhang CL Yang D. Effects of nine steaming-nine drying on the physicochemical properties and antioxidant activity of *Polygonatum sibiricum* red. Mod Food Sci Technol. (2024) 40:231–45. doi: 10.13982/j.mfst.1673-9078.2024.2.0036

[26] Wu LL Huang NW Xiong MH Huang JP Zhao HJ Xie W. Comparative study on the effects of nine-steaming and nine-system process on the functional components of three types of Polygonatum. Technol Innov Appl. (2023) 13:49–53. doi: 10.19981/j.CN23-1581/G3.2023.20.011

[27] Wang D Shi LJ Fan XW Luo HQ Li WT Li YL. Development and validation of an efficient HILIC-QQQ-MS/MS method for quantitative and comparative profiling of 45 hydrophilic compounds in four types of tea (Camellia sentences). Food Chem. (2022) 371:131201. doi: 10.1016/j.foodchem.2021.131201, PMID: 34598116

[28] Hu Y Yin MZ Bai YJ Chu SS Zhang L Yang M. An evaluation of traits, nutritional, and medicinal component quality of *Polygonatum cyrtonema* Hua and *P. sibiricum* red. Front Plant Sci. (2022) 13:891775. doi: 10.3389/fpls.2022.891775, PMID: 35519815 PMC9062581

[29] Guan YH Liang ZW Shi Y Xu SS Liao Q Wang T. Differences in chemical constituents of *Polygonatum kingianum* before and after processing. Chin TraditHerb Drugs. (2024) 11:3647–58. doi: 10.7501/j.issn.0253-2670.2024.11.008

[30] Qu FF Zeng WC Tong X Feng W Chen YQ Ni DJ. The new insight into the influence of fermentation temperature on quality and bioactivities of black tea. LWT. (2020) 117:108646. doi: 10.1016/j.lwt.2019.108646

[31] Tao H Li LY He YQ Zhang XY Zhao Y Wang QM. Flavonoids in vegetables: improvement of dietary flavonoids by metabolic engineering to promote health. Crit Rev Food Sci Nutr. (2024) 64:3220–34. doi: 10.1080/10408398.2022.2131726, PMID: 36218329

[32] Ren ZT Yin X Liu LP Zhang L Shen WJ Fang ZX. Flavonoid localization in soybean seeds: comparative analysis of wild (*Glycine soja*) and cultivated (*Glycine max*) varieties. Food Chem. (2024) 456:139883. doi: 10.1016/j.foodchem.2024.139883, PMID: 38870803

[33] Miranda RS de Jesus BDSM da Silva Luiz SR Viana CB Adão Malafaia CR Figueiredo FS. Antiinflammatory activity of natural triterpenes-an overview from 2006 to 2021. Phytother Res. (2022) 36:1459–506. doi: 10.1002/ptr.7359, PMID: 35229374

[34] Timilsena YP Phosanam A Stockmann R. Perspectives on Saponins: food functionality and applications. Int J Mol Sci. (2023) 24:13538. doi: 10.3390/ijms241713538, PMID: 37686341 PMC10487995

[35] Chen SY Wang L Lyu Q Shan QY Han X Yang Q. Molecular network strategies combined with MCnebula2 identify potential active compounds from steamed *Polygonatum cyrtonema* Hua. J Chromatogr A. (2025) 1746:465779. doi: 10.1016/j.chroma.2025.465779, PMID: 39983564

[36] Wang C Ye J He XG Tang YY. Research progress on chemical components and pharmacological effects of Polygonati Rhizoma and prediction analysis of quality marker. Nat Prod Res Dev. (2024) 36:881–899+855. doi: 10.16333/j.1001-6880.2024.5.017

[37] Singh B Singh JP Singh N Kaur A. Saponins in pulses and their health promoting activities: a review. Food Chem. (2017) 233:540–9. doi: 10.1016/j.foodchem.2017.04.161, PMID: 28530610

[38] Sun CY Li YT Liu D Chen CW Liao ML. Gastroprotective potential of the aqueous extract of nine-steaming and nine-sun-drying processed *Polygonatum cyrtonema* Hua against alcoholic gastric injury in mice. J Ethnopharmacol. (2025) 338:119103. doi: 10.1016/j.jep.2024.119103, PMID: 39542190

[39] Luo WG Li XW Zhang CX Shen K Li MX Zhuang Y. Physicochemical characterization and protective effects of raw and nine-steamed *Polygonatum cyrtonema* polysaccharides on cyclophosphamide-induced immunosuppression in mice. Int J Biol Macromol. (2025) 307:141911. doi: 10.1016/j.ijbiomac.2025.141911 40068755

[40] Yao XJ Zhang YX Zhang B Deng ZY Li HY. The structure change of Polygonatum polysaccharide and the protect effect of *Polygonatum cyrtonema* Hua extracts and polysaccharide on cisplatin-induced AKI mice during nine-steam-nine-bask processing. Int J Biol Macromol. (2024) 277:132290. doi: 10.1016/j.ijbiomac.2024.132290 38795899

[41] Wang XL Cheng YN Zheng B Chen Y Xie JH Hu X. Effects of nine-steam-nine-bask processing on the bioactive compounds content, bioaccessibility, and antioxidant capacity of *Polygonatum cyrtonema* Hua. J Funct Foods. (2024) 117:106236. doi: 10.1016/j.jff.2024.106236, PMID: 40151496

[42] Zhang QH Lin XY Su WK. Study on the components changes of polysaccharides and saponins during nine steaming and drying of *Polygonatum sibiricum*. J Sci Food Agric. (2024) 104:6862–74. doi: 10.1002/jsfa.13516 38587108

[43] Fan XY Zhou L Xing YC Wang LM Choi SS Zhang Z. A comprehensive investigation on the chemical changes of traditional Chinese medicine with classic processing technology: *Polygonum multiflorum* under nine cycles of steaming and sunning as a case study. Anal Bioanal Chem. (2024) 416:1733–44. doi: 10.1007/s00216-024-05177-0, PMID: 38347251

[44] Dong HY Li YH Lai XF Hao MJ Sun LL Li QH. Effects of fermentation duration on the flavour quality of large leaf black tea based on metabolomics. Food Chem. (2024) 444:138680. doi: 10.1016/j.foodchem.2024.138680, PMID: 38325077

[45] Nie JL Fu XT Wang L Xu JC Gao X. Impact of Monascus purpureus fermentation on antioxidant activity, free amino acid profiles and flavor properties of kelp (Saccharina japonica). Food Chem. (2023) 400:133990. doi: 10.1016/j.foodchem.2022.133990, PMID: 36063678

[46] Hou Y Mao HL Lu FM Ma CQ Zhu SX Li GY. Widely targeted metabolomics and HPLC analysis elaborated the quality formation of Yunnan pickled tea during the whole process at an industrial scale. Food Chem. (2023) 422:135716. doi: 10.1016/j.foodchem.2023.135716, PMID: 37156017

[47] Bork LV Stobernack T Rohn S Kanzler C. Browning reactions of hydroxycinnamic acids and heterocyclic Maillard reaction intermediates - formation of phenol-containing colorants. Food Chem. (2024) 449:139189. doi: 10.1016/j.foodchem.2024.139189, PMID: 38593726

[48] Wang S Wang LL Fang JJ Liu KL Wang YZ Zhang C. Correlation analysis between color and content changes of five components of wine-processed *Polygonatum kingianum* rhizoma during processing. Chin J Exp Tradit Med Formulae. (2022) 28:156–62. doi: 10.13422/j.cnki.syfjx.20220252

