# Supplementary material for: Non-targeted metabolomics-mediated elucidation of metabolite changes in Polygonatum kingianum during traditional steaming with black beans
Source: Front Nutr. 2025 Apr 11;12:1581459. doi: 10.3389/fnut.2025.1581459 (PMC12021634; doi:10.3389/fnut.2025.1581459)
Supplement: Supplementary file 1 [file Data_Sheet_1.ZIP › Supplementary-Material.docx]

Supplementary Material

1. **Supplementary Tables**

|  | L* | a* | b* | C* | h° | Eab* |
| --- | --- | --- | --- | --- | --- | --- |
| S | 92.92±0.10^Aa^ | 0.45±0.04^Gg^ | 10.33±0.14^CDd^ | 10.33±0.14^DEe^ | 87.48±0.22^Aa^ | 93.50±0.10^Aa^ |
| P1 | 81.92±0.43^Bb^ | 3.96±0.21^Ff^ | 17.16±0.02^Bb^ | 17.62±0.04^Bb^ | 77.00±0.66^Bb^ | 83.79±0.42^Bb^ |
| P2 | 61.17±1.47^Cc^ | 12.43±0.33^Aa^ | 24.16±0.48^Aa^ | 27.17±0.53^Aa^ | 62.77±0.53^Cc^ | 66.93±1.53^Cc^ |
| P3 | 49.23±2.92^Dd^ | 9.81±0.48^Bb^ | 11.88±0.99^Cc^ | 15.4±1.05^Cc^ | 50.47±1.27^Dde^ | 51.59±2.98^Dd^ |
| P4 | 45.67±0.65^Ee^ | 7.75±0.66^Cc^ | 8.94±0.90^Dde^ | 11.83±1.11^Dd^ | 49.07±0.60^Dde^ | 47.18±0.92^Ee^ |
| P5 | 45.31±1.32^Ee^ | 7.12±0.46^CcDd^ | 7.87±0.32^DEe^ | 10.61±0.52^DdEe^ | 47.83±1.13^De^ | 46.54±1.39^Ee^ |
| P6 | 44.90±0.64^Ee^ | 6.59±0.11^Dd^ | 7.56±0.06^DEe^ | 10.03±0.04^DEef^ | 48.94±0.69^Dde^ | 46.01±0.63^Ee^ |
| P7 | 44.41±0.92^Ee^ | 6.04±0.52^DdEe^ | 7.03±0.66^DEe^ | 9.27±0.84^Eef^ | 49.34±0.26^Dde^ | 45.37±1.07^Ee^ |
| P8 | 43.96±1.33^Ee^ | 5.88±0.18^DEe^ | 6.98±1.05^DEe^ | 9.01±0.95^Eef^ | 50.75±2.98^Dde^ | 44.90±1.45^Ee^ |
| P9 | 43.70±3.27^Ee^ | 5.58±0.26^Ee^ | 6.89±1.92^Ee^ | 8.68±1.74^Ef^ | 52.54±5.86^Dd^ | 44.64±2.93^Ee^ |

**Table S1.** Results of color determination of PK.

Notes: different capital letters in each column indicate significance *P* < 0.01, and different lowercase letters indicate *P* < 0.05. Data are expressed as the mean ± standard deviation (n = 3).

**Table S2.** One-way analysis of variance (ANOVA) results of the main quality components of nine-steamed and nine-processed PK (%).

|  | saponin | polysaccharide | reducing sugar | Total flavonoids |
| --- | --- | --- | --- | --- |
| S | 4.79±0.05^Ccd^ | 17.70±0.17^Aa^ | 0.17±0.00^Ii^ | 0.033±0.004^Gh^ |
| P1 | 3.84±0.10^De^ | 13.59±0.00^Bb^ | 3.62±0.03^Hh^ | 0.043±0.004^Gh^ |
| P2 | 4.89±0.10^Cc^ | 13.56±0.33^Bb^ | 20.02±0.16^Dd^ | 0.104±0.004^Fg^ |
| P3 | 5.81±0.10^Aa^ | 10.03±0.17^Cc^ | 27.36±0.10^Aa^ | 0.274±0.006^De^ |
| P4 | 5.66±0.10^Aa^ | 9.78±0.00^Cc^ | 23.68±0.00^Bb^ | 0.404±0.009^Cd^ |
| P5 | 5.28±0.05^Bb^ | 7.96±0.08^Ff^ | 23.49±0.10^Bb^ | 0.531±0.006^Ab^ |
| P6 | 4.71±0.15^Ccd^ | 8.74±0.00^Dd^ | 20.85±0.26^Cc^ | 0.551±0.004^Aa^ |
| P7 | 4.72±0.15^Ccd^ | 8.33±0.08^Ee^ | 18.47±0.16^Ee^ | 0.435±0.015^Bc^ |
| P8 | 4.61±0.10^Cd^ | 8.98±0.00^Dd^ | 15.61±0.20^Ff^ | 0.425±0.002^Bc^ |
| P9 | 2.75±0.05^Ef^ | 8.74±0.17^Dd^ | 10.54±0.07^Gg^ | 0.228±0.000^Ef^ |

Notes: different capital letters in each column indicate significance *P* < 0.01, and different lowercase letters indicate *P* < 0.05. Data are expressed as the mean ± standard deviation (n = 3).

**Table S3.** Comparison of up- and down-regulated differential metabolites in 10 groups of samples.

| **Compare** | **Up**  **（**VIP>1.0，P<0.05，FC>1.0**）** | **Down**  **（**VIP>1.0，P<0.05，FC＜1.0**）** | **Sum** |
| --- | --- | --- | --- |
| P2-vs-P1 | 278 | 208 | 486 |
| P3-vs-P2 | 357 | 128 | 485 |
| P4-vs-P3 | 152 | 213 | 365 |
| P5-vs-P4 | 310 | 166 | 476 |
| P6-vs-P5 | 61 | 121 | 182 |
| P7-vs-P6 | 89 | 169 | 258 |
| P8-vs-P7 | 128 | 61 | 189 |
| P9-vs-P8 | 79 | 101 | 180 |
| P1-vs-S | 158 | 121 | 279 |
| P2-vs-S | 236 | 161 | 397 |
| P3-vs-S | 288 | 165 | 453 |
| P4-vs-S | 289 | 176 | 465 |
| P5-vs-S | 310 | 177 | 487 |
| P6-vs-S | 305 | 179 | 484 |
| P7-vs-S | 286 | 179 | 465 |
| P8-vs-S | 279 | 179 | 458 |
| P9-vs-S | 280 | 177 | 457 |

**Table S4.** Differential metabolite pathway enrichment information.

| **ID** | **Pathway** | **p-value** | **Metabolites (KEGG number)** | **Number of metabolites enriched** |
| --- | --- | --- | --- | --- |
| osa00940 | Phenylpropanoid biosynthesis | 0.001462 | C00082, C00852, C01175, C01494, C01752, C01772, C05619, C12205 | 8 |
| osa00670 | One carbon pool by folate | 0.002468 | C00101, C00440, C00504 | 3 |
| osa00941 | Flavonoid biosynthesis | 0.002531 | C00509, C00852, C01604, C06562, C09762, C09789, C09833, C12123 | 8 |
| osa00740 | Riboflavin metabolism | 0.003489 | C01007, C01847, C04454, C18910 | 4 |
| osa00220 | Arginine biosynthesis | 0.005912 | C00049, C00062, C00327, C00624 | 4 |
| osa00965 | Betalain biosynthesis | 0.006918 | C00082, C00355, C03758, C17755 | 4 |
| osa00330 | Arginine and proline metabolism | 0.012855 | C00062, C00436, C03166, C04281, C05932, C05938, C05945 | 7 |
| osa00350 | Tyrosine metabolism | 0.012855 | C00082, C00355, C00530, C03758, C04368, C04797, C05596 | 7 |
| osa00380 | Tryptophan metabolism | 0.017678 | C00643, C00954, C02220, C03453, C03824, C05660, C05834 | 7 |
| osa00030 | Pentose phosphate pathway | 0.025899 | C00198, C01151, C01801, C06473 | 4 |
| osa00970 | Aminoacyl-tRNA biosynthesis | 0.02607 | C00049, C00062, C00082, C00101, C00135 | 5 |
| osa00460 | Cyanoamino acid metabolism | 0.057574 | C00049, C00082, C01594, C08325 | 4 |
| osa00052 | Galactose metabolism | 0.061529 | C00137, C00795, C00880, C05396 | 4 |
| osa00340 | Histidine metabolism | 0.065625 | C00049, C00135, C00388, C20522 | 4 |
| osa00770 | Pantothenate and CoA biosynthesis | 0.072694 | C00049, C00099, C05944 | 3 |
| osa00310 | Lysine degradation | 0.078744 | C00739, C03087, C03656, C05545 | 4 |
| osa02010 | ABC transporters | 0.081608 | C00009, C00049, C00062, C00135, C00137, C00212 | 6 |
| osa00410 | beta-Alanine metabolism | 0.084793 | C00049, C00099, C00135 | 3 |
| osa00564 | Glycerophospholipid metabolism | 0.088165 | C00157, C00350, C01233, C04230 | 4 |
| osa04136 | Autophagy - other | 0.094805 | C00350 | 1 |
| osa00400 | Phenylalanine, tyrosine and tryptophan biosynthesis | 0.09769 | C00082, C00296, C16850 | 3 |
| osa00261 | Monobactam biosynthesis | 0.133054 | C00049, C00062, C00082 | 3 |
| osa00270 | Cysteine and methionine metabolism | 0.148732 | C00049, C00170, C01077, C11499 | 4 |
| osa00232 | Caffeine metabolism | 0.160137 | C16352, C16364 | 2 |
| osa00290 | Valine, leucine and isoleucine biosynthesis | 0.171832 | C02504, C04181 | 2 |
| osa00592 | alpha-Linolenic acid metabolism | 0.172145 | C00157, C04780, C16316 | 3 |
| osa00600 | Sphingolipid metabolism | 0.195613 | C01190, C12144 | 2 |
| osa00053 | Ascorbate and aldarate metabolism | 0.222674 | C00137, C00818, C03033 | 3 |
| osa00591 | Linoleic acid metabolism | 0.231956 | C00157, C14767 | 2 |
| osa04075 | Plant hormone signal transduction | 0.258488 | C00954 | 1 |
| osa00760 | Nicotinate and nicotinamide metabolism | 0.266764 | C00049, C01004, C05380 | 3 |
| osa00195 | Photosynthesis | 0.306237 | C00009 | 1 |
| osa00360 | Phenylalanine metabolism | 0.3118 | C00082, C01772, C05852 | 3 |
| osa00500 | Starch and sucrose metabolism | 0.341801 | C03323, C16688 | 2 |
| osa00785 | Lipoic acid metabolism | 0.350936 | C16238 | 1 |
| osa00908 | Zeatin biosynthesis | 0.365742 | C00170, C04713 | 2 |
| osa00190 | Oxidative phosphorylation | 0.412683 | C00009 | 1 |
| osa00563 | Glycosylphosphatidylinositol (GPI)-anchor biosynthesis | 0.412683 | C00350 | 1 |
| osa00590 | Arachidonic acid metabolism | 0.445987 | C00157, C05962, C06462 | 3 |
| osa00640 | Propanoate metabolism | 0.468625 | C00099, C04593 | 2 |
| osa00520 | Amino sugar and nucleotide sugar metabolism | 0.472445 | C00203, C00329, C02336, C04089 | 4 |
| osa00260 | Glycine, serine and threonine metabolism | 0.490158 | C00049, C00101 | 2 |
| osa00430 | Taurine and hypotaurine metabolism | 0.519235 | C05122 | 1 |
| osa00710 | Carbon fixation in photosynthetic organisms | 0.535027 | C00049 | 1 |
| osa00565 | Ether lipid metabolism | 0.565082 | C01233 | 1 |
| osa00945 | Stilbenoid, diarylheptanoid and gingerol biosynthesis | 0.565082 | C00852 | 1 |
| osa00790 | Folate biosynthesis | 0.570647 | C00101, C00504 | 2 |
| osa00902 | Monoterpenoid biosynthesis | 0.580047 | C00400, C01512 | 2 |
| osa00073 | Cutin, suberine and wax biosynthesis | 0.59321 | C19620 | 1 |
| osa00250 | Alanine, aspartate and glutamate metabolism | 0.60659 | C00049 | 1 |
| osa04070 | Phosphatidylinositol signaling system | 0.619533 | C00137 | 1 |
| osa00240 | Pyrimidine metabolism | 0.633293 | C00099, C01168 | 2 |
| osa00620 | Pyruvate metabolism | 0.644166 | C02504 | 1 |
| osa00730 | Thiamine metabolism | 0.644166 | C00082 | 1 |
| osa00130 | Ubiquinone and other terpenoid-quinone biosynthesis | 0.681177 | C00082, C05817 | 2 |
| osa00300 | Lysine biosynthesis | 0.688786 | C00049 | 1 |
| osa00480 | Glutathione metabolism | 0.718567 | C01879 | 1 |
| osa00650 | Butanoate metabolism | 0.753923 | C00497 | 1 |
| osa00950 | Isoquinoline alkaloid biosynthesis | 0.769177 | C00082, C00355, C03758 | 3 |
| osa00909 | Sesquiterpenoid and triterpenoid biosynthesis | 0.784381 | C06080, C09627 | 2 |
| osa00562 | Inositol phosphate metabolism | 0.791983 | C00137 | 1 |
| osa00944 | Flavone and flavonol biosynthesis | 0.818177 | C04608 | 1 |
| osa00230 | Purine metabolism | 0.824151 | C00212, C00387 | 2 |
| osa00440 | Phosphonate and phosphinate metabolism | 0.83002 | C01151 | 1 |
| osa00051 | Fructose and mannose metabolism | 0.835652 | C01355 | 1 |
| osa00040 | Pentose and glucuronate interconversions | 0.846365 | C03033 | 1 |
| osa00630 | Glyoxylate and dicarboxylate metabolism | 0.874523 | C00988 | 1 |
| osa00942 | Anthocyanin biosynthesis | 0.890387 | C12141 | 1 |
| osa00960 | Tropane, piperidine and pyridine alkaloid biosynthesis | 0.897556 | C01479 | 1 |
| osa00966 | Glucosinolate biosynthesis | 0.924475 | C00082 | 1 |
| osa00998 | Biosynthesis of various antibiotics | 0.934061 | C00082 | 1 |
| osa00906 | Carotenoid biosynthesis | 0.97861 | C05431 | 1 |
| osa00860 | Porphyrin metabolism | 0.990631 | C02800 | 1 |

**Table S5.** Overall expression abundance (×10^6^) and one-way ANOVA for several major classes of differential metabolites.

|  | S | P1 | P2 | P3 | P4 | P5 | P6 | P7 | P8 | P9 |
| --- | --- | --- | --- | --- | --- | --- | --- | --- | --- | --- |
| Fatty Acyls，Steroids and steroid derivatives | 77.74±5.89^Aa^ | 65.39±17.80^Aa^ | 35.33±2.21^Bb^ | 32.97±2.05^Bb^ | 30.35±3.08^Bb^ | 30.60±6.44^Bb^ | 29.25±3.22^Bb^ | 29.91±0.56^Bb^ | 32.91±3.63^Bb^ | 32.17±2.31^Bb^ |
| Amino acids, peptides, and analogues | 109.06±20.90^Aa^ | 104.20±16.37^AaBb^ | 89.33±12.41^ABb^ | 84.78±5.89^Bb^ | 63.30±4.48^BCc^ | 54.46±1.52^Ccd^ | 52.33±1.00^Ccd^ | 40.59±3.18^Cd^ | 41.28±2.53^Cd^ | 40.30±2.24^Cd^ |
| Carbohydrates and carbohydrate conjugates | 92.02±34.38^ABb^ | 93.99±13.85^ABb^ | 101.21±16.77^AaBb^ | 124.25±11.40^Aa^ | 86.92±4.61^Bb^ | 90.14±6.88^ABb^ | 92.08±15.27^ABb^ | 78.09±13.54^Bb^ | 70.40±5.53^Bb^ | 70.64±7.25^Bb^ |
| Organic acids and derivatives | 29.55±8.41^Bc^ | 31.64±2.67^Bb^ | 36.21±1.58^ABb^ | 44.07±1.84^Aa^ | 39.46±2.66^AaBb^ | 41.86±2.82^Aab^ | 37.93±3.40^AaBb^ | 30.85±2.87^Bb^ | 31.61±2.40^Bb^ | 30.68±2.57^Bb^ |
| Flavonoids and derivatives | 10.79±3.01^Bb^ | 11.33±1.50^Ab^ | 14.09±4.01^Aab^ | 15.62±0.57^Aa^ | 13.74±0.44^Aab^ | 14.03±0.39^Aab^ | 15.38±2.30^Aa^ | 11.99±1.62^Ab^ | 11.44±0.78^Ab^ | 11.55±1.35^Ab^ |
| Nucleosides, nucleotides, and analogues | 5.10±2.67^AaBb^ | 7.40±4.76^Aa^ | 7.09±1.95^AaBb^ | 4.78±0.20^AaBb^ | 3.99±0.33^ABb^ | 3.90±0.22^ABb^ | 3.70±0.27^ABb^ | 3.19±0.31^ABb^ | 2.88±0.22^Bb^ | 2.86±0.31^Bb^ |
| Cinnamaldehydes,Cinnamic acids and derivatives | 21.09±15.20^Aa^ | 17.47±11.88^AaBb^ | 8.97±2.15^ABb^ | 5.40±0.26^Bb^ | 4.50±0.07^Bb^ | 4.57±0.26^Bb^ | 4.67±0.28^Bb^ | 4.57±0.41^Bb^ | 3.90±0.16^Bb^ | 4.00±0.52^Bb^ |
| Coumarins,Isocoumarins and derivatives | 3.31±0.76^Aa^ | 1.38±0.24^Bc^ | 2.10±0.30^Bb^ | 3.27±0.37^Aa^ | 3.48±0.39^Aa^ | 3.57±0.23^Aa^ | 3.67±0.23^Aa^ | 3.33±0.25^Aa^ | 3.66±0.08^Aa^ | 3.83±0.53^Aa^ |
| Lignans, neolignans and related compounds | 0.02±0.02^Dd^ | 0.10±0.04^Cc^ | 0.17±0.01^Bb^ | 0.26±0.02^Aa^ | 0.24±0.02^AaBb^ | 0.24±0.01^AaBb^ | 0.24±0.01^AaBb^ | 0.21±0.02^Bb^ | 0.19±0.02^Bb^ | 0.20±0.01^Bb^ |
| Chalcones and dihydrochalcones | 0.06±0.10^Dd^ | 2.62±0.59^Aa^ | 1.63±0.40^Bb^ | 0.96±0.04^Cc^ | 0.82±0.06^Cc^ | 1.10±0.06^BCc^ | 1.24±0.41^BbCc^ | 0.94±0.26^Cc^ | 0.80±0.10^Cc^ | 0.80±0.15^Cc^ |
| Alkaloids and derivatives | 1.37±1.00^Aab^ | 1.02±0.10^Ab^ | 1.21±0.25^Ab^ | 1.59±0.21^Aab^ | 1.72±0.12^Aab^ | 1.61±0.15^Aab^ | 1.82±0.30^Aa^ | 1.32±0.08^Aab^ | 1.39±0.04^Aab^ | 1.43±0.10^Aab^ |
| Phenols | 0.89±0.33^Cc^ | 2.44±0.47^Aa^ | 2.52±0.20^Aa^ | 2.36±0.08^Aab^ | 2.07±0.03^ABb^ | 1.88±0.01^Bb^ | 2.07±0.08^ABb^ | 1.89±0.10^Bb^ | 1.74±0.08^Bb^ | 1.77±0.09^Bb^ |
| Total | 350.98±77.12^Aa^ | 339.01±23.57^Aa^ | 299.86±24.82^AaBb^ | 320.31±18.81^AaB^ | 250.60±12.52^Bb^ | 247.97±11.43^Bb^ | 244.38±23.16^Bb^ | 206.88±22.11^Bb^ | 202.20±9.12^Bb^ | 200.23±14.25^Bb^ |

Notes: different capital letters in each column indicate significance *P* < 0.01, and different lowercase letters indicate *P* < 0.05. Data are expressed as the mean ± standard deviation (n = 3).

**Table S6.** Mean expression abundance of 25 saponins produced and significantly accumulated after steaming (×10^3^).

| **Class** | **Metabolites** | **S** | **P1** | **P2** | **P3** | **P4** | **P5** | **P6** | **P7** | **P8** | **P9** |
| --- | --- | --- | --- | --- | --- | --- | --- | --- | --- | --- | --- |
| Triterpenoid saponin | Theasaponin E5 | / | / | 769.64 | 2098.03 | 1706.05 | 1089.08 | 821.09 | 652.62 | 830.08 | 624.80 |
|  | Acutoside A | / | 0.34 | 22.68 | 6.97 | 14.01 | 7.98 | 9.35 | 13.30 | 23.24 | 24.05 |
|  | Astragaloside Iii | / | / | / | 0.19 | 0.71 | 1.02 | 2.49 | 4.41 | 10.88 | 15.77 |
|  | Hoduloside Iii | / | 4.21 | 159.46 | 59.79 | 144.37 | 83.67 | 94.82 | 139.30 | 193.02 | 205.57 |
|  | Lucyoside L | / | / | 14.13 | 197.56 | 220.97 | 275.37 | 187.28 | 129.87 | 105.56 | 143.07 |
|  | Pisumsaponin I | / | 12.05 | 213.80 | 98.89 | 165.45 | 126.43 | 130.06 | 180.15 | 222.29 | 233.15 |
|  | Pisumsaponin Ii | / | / | 13.44 | 3.01 | 10.08 | 5.43 | 8.37 | 16.35 | 31.50 | 33.68 |
|  | Sandosaponin A | / | 0.52 | 37.49 | 11.21 | 27.69 | 16.97 | 19.68 | 27.03 | 40.85 | 46.18 |
|  | Soyasapogenol B 3-O-[A-L-Rhamnosyl-(1->4)-B-D-Galactosyl-(1->4)-B-D-Glucuronide] | / | 76.02 | 1630.10 | 683.80 | 1286.94 | 947.37 | 1005.88 | 1446.07 | 1966.86 | 2049.75 |
|  | Soyasapogenol B 3-O-B-D-Glucuronide | / | / | 12.36 | 5.33 | 17.94 | 14.61 | 22.82 | 37.67 | 80.80 | 105.62 |
|  | Soyasaponin Ii | / | 24.22 | 565.37 | 233.76 | 505.02 | 353.23 | 376.15 | 598.93 | 928.73 | 992.50 |
|  | Soyasaponin Iii | / | 0.40 | 36.36 | 9.44 | 21.95 | 13.77 | 14.94 | 24.37 | 43.32 | 47.01 |
|  | Soyasaponin V | / | 23.59 | 423.37 | 151.41 | 298.60 | 186.79 | 201.89 | 298.08 | 437.53 | 488.44 |
|  | Pitheduloside A | / | 1.06 | 56.51 | 19.67 | 46.64 | 34.69 | 42.03 | 74.97 | 148.67 | 175.54 |
|  | Cyclopassifloside Vii | 6.90 | 53.18 | 7.68 | 21.65 | 8.59 | 8.58 | 29.95 | 25.59 | 12.68 | 11.64 |
| Steroidal saponin | Trillfurostanoside F | / | 0.05 | 30.68 | 13.52 | 28.36 | 22.50 | 28.29 | 41.02 | 69.16 | 81.15 |
|  | Yuccoside C | / | 576.67 | 8.30 | 12.52 | 2.45 | 0.36 | 0.23 | / | 0.35 | 1.05 |
|  | Parisvanioside E | 0.04 | 2145.55 | 1186.19 | 1203.76 | 786.25 | 264.44 | 113.98 | 74.28 | 65.17 | 56.74 |
|  | Chinenoside V | 34.58 | 386.64 | 286.49 | 464.36 | 219.23 | 102.54 | 206.27 | 224.05 | 107.65 | 189.90 |
|  | Isonuatigenin 3-[Rhamnosyl-(1->2)-Glucoside] | 328.58 | 2714.68 | 296.25 | 1063.51 | 1306.35 | 746.31 | 735.22 | 939.79 | 908.95 | 1074.75 |
| Steroidogenic saponin | Asparasaponin Ii | / | 261.14 | 3.96 | 6.58 | 1.69 | 1.67 | 0.22 | / | 0.20 | 0.12 |
|  | Fevicordin B 2-[Rhamnosyl-(1->4)-Glucosyl-(1->6)-Glucoside] | / | 336.89 | 184.60 | 194.78 | 110.94 | 36.93 | 13.73 | 7.39 | 5.83 | 4.35 |
|  | Evasterioside E | 0.87 | 660.73 | 25.68 | 22.55 | 13.90 | 3.71 | 3.43 | 2.37 | 0.74 | 1.27 |
|  | Melongoside O | 17.27 | 3926.43 | 264.64 | 206.03 | 81.81 | 44.43 | 28.84 | 28.37 | 12.38 | 21.41 |
|  | Melongoside F | 146.99 | 1834.66 | 272.51 | 1206.15 | 1534.87 | 971.40 | 909.01 | 1167.63 | 1049.80 | 1350.10 |

1. **Supplementary Figures**


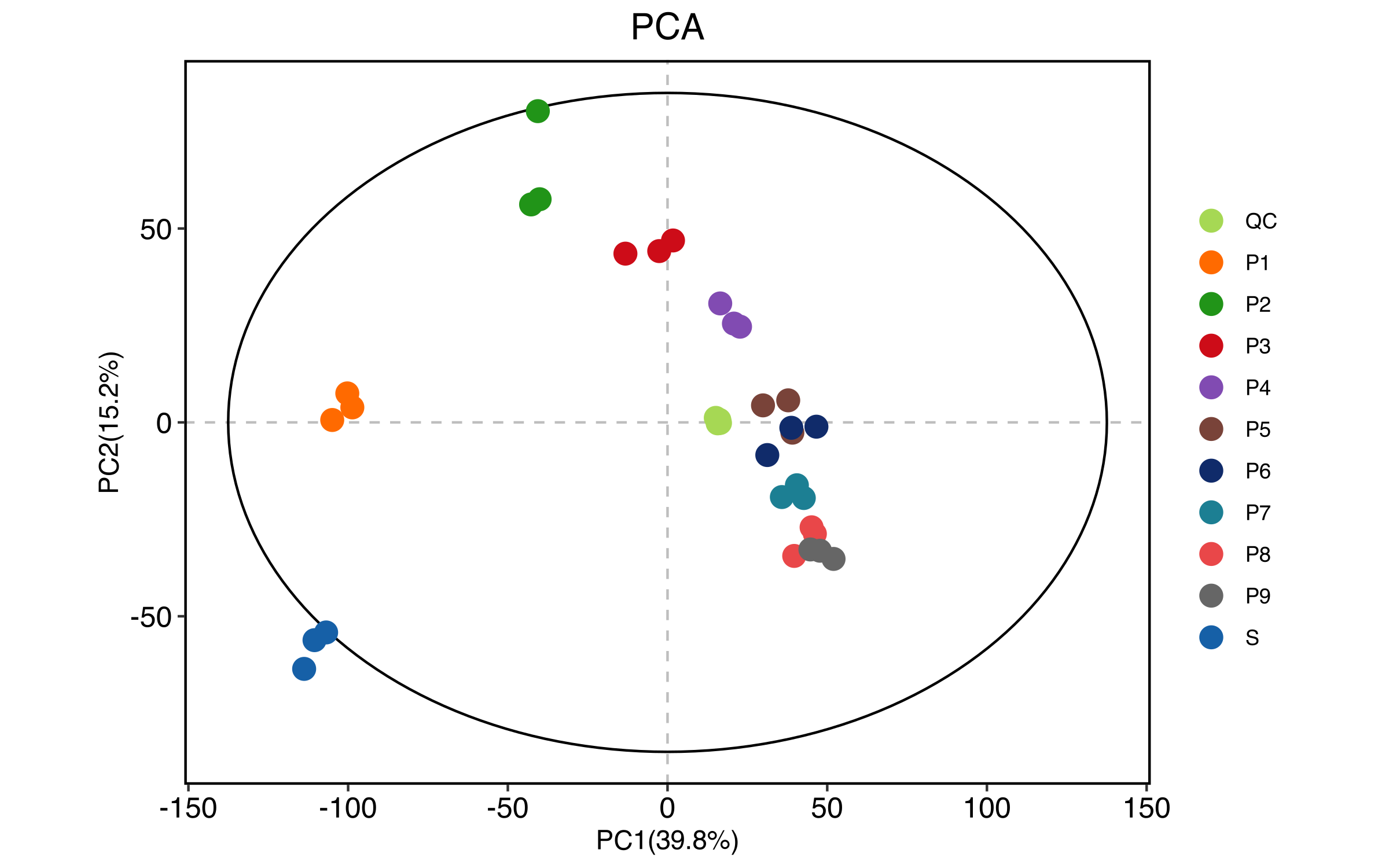


**Figure S1.** PCA distribution diagram including quality control samples.


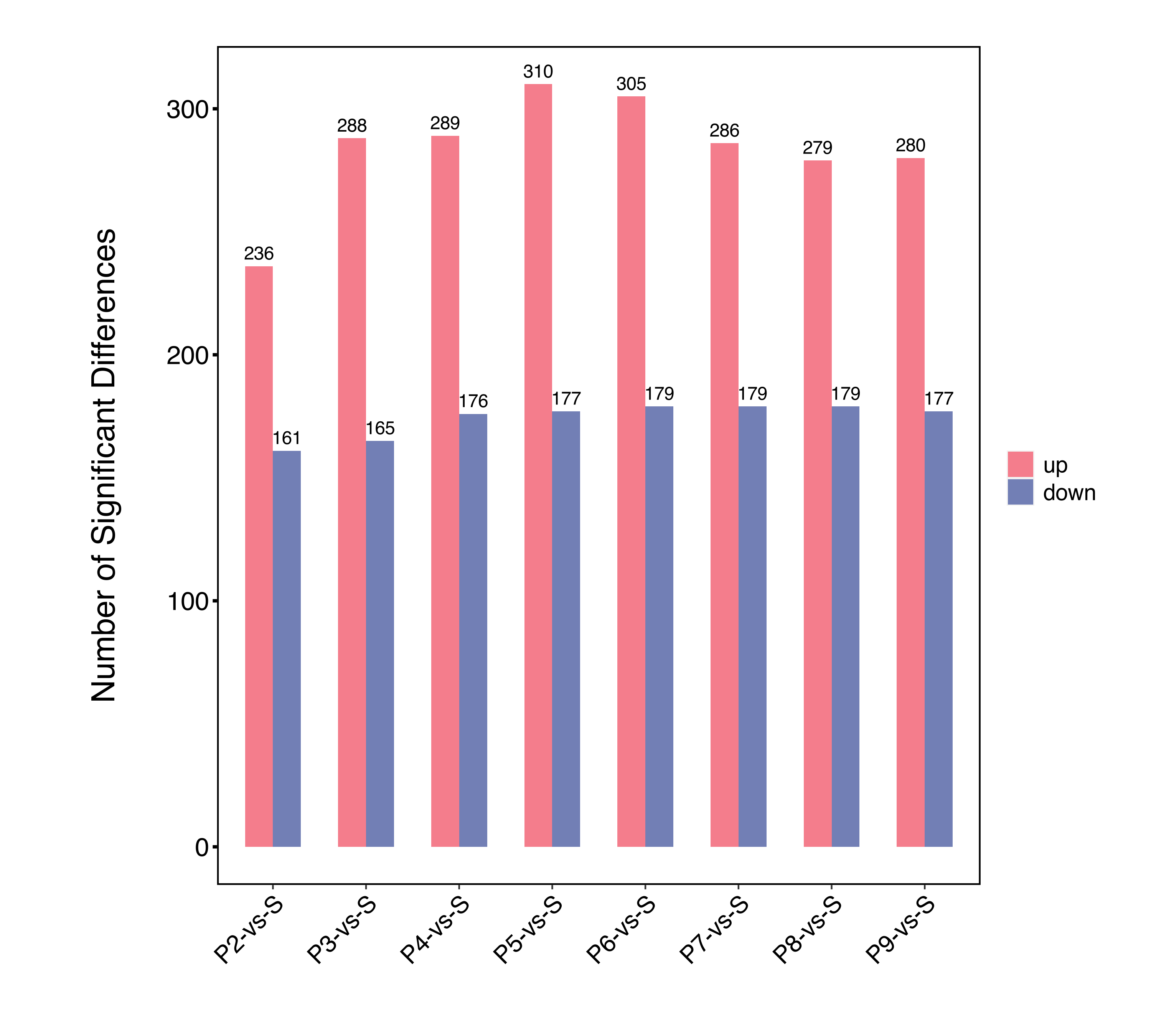

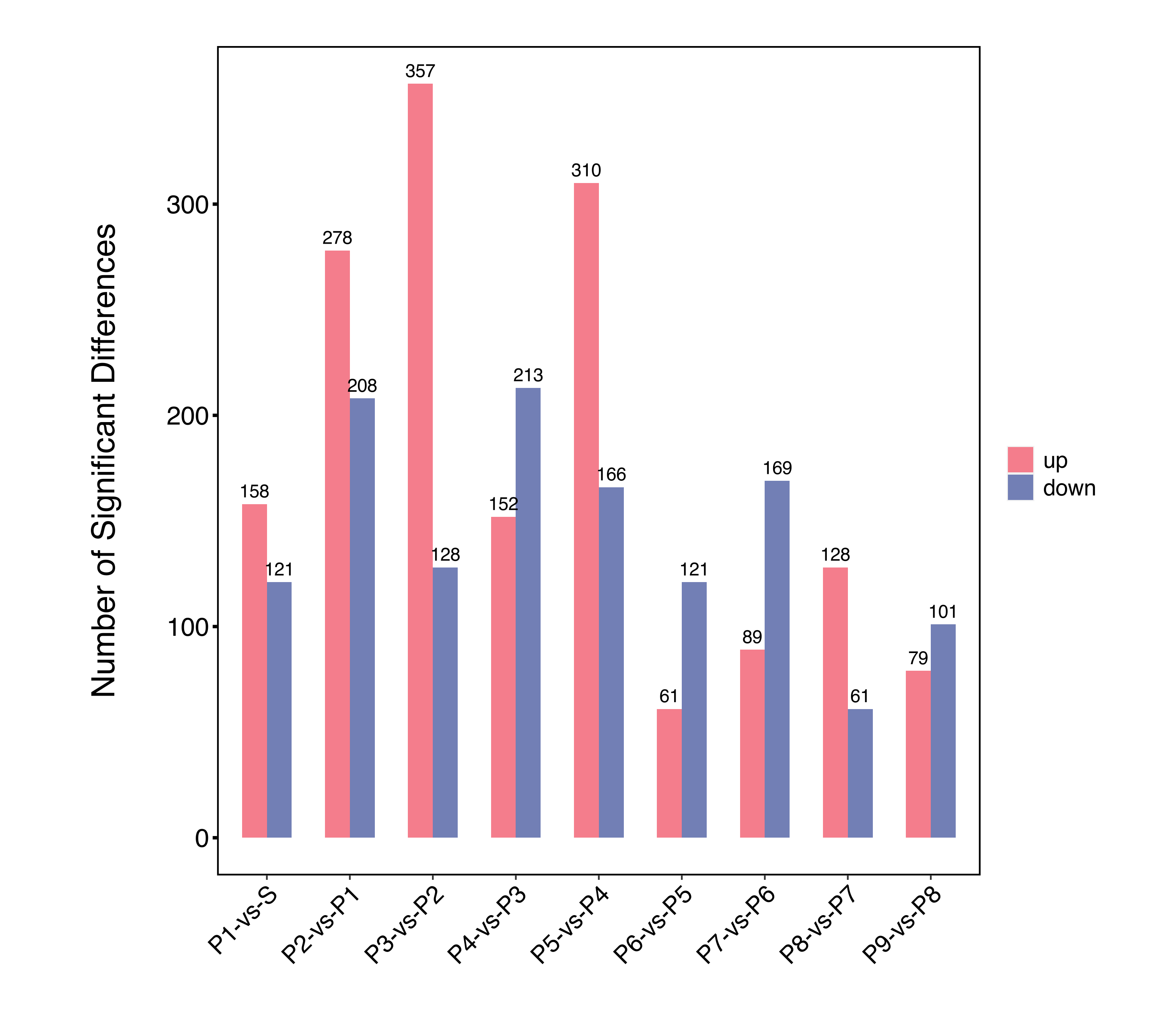


**Figure S2.** Comparison of up- and down-regulated metabolites in 10 groups of samples.


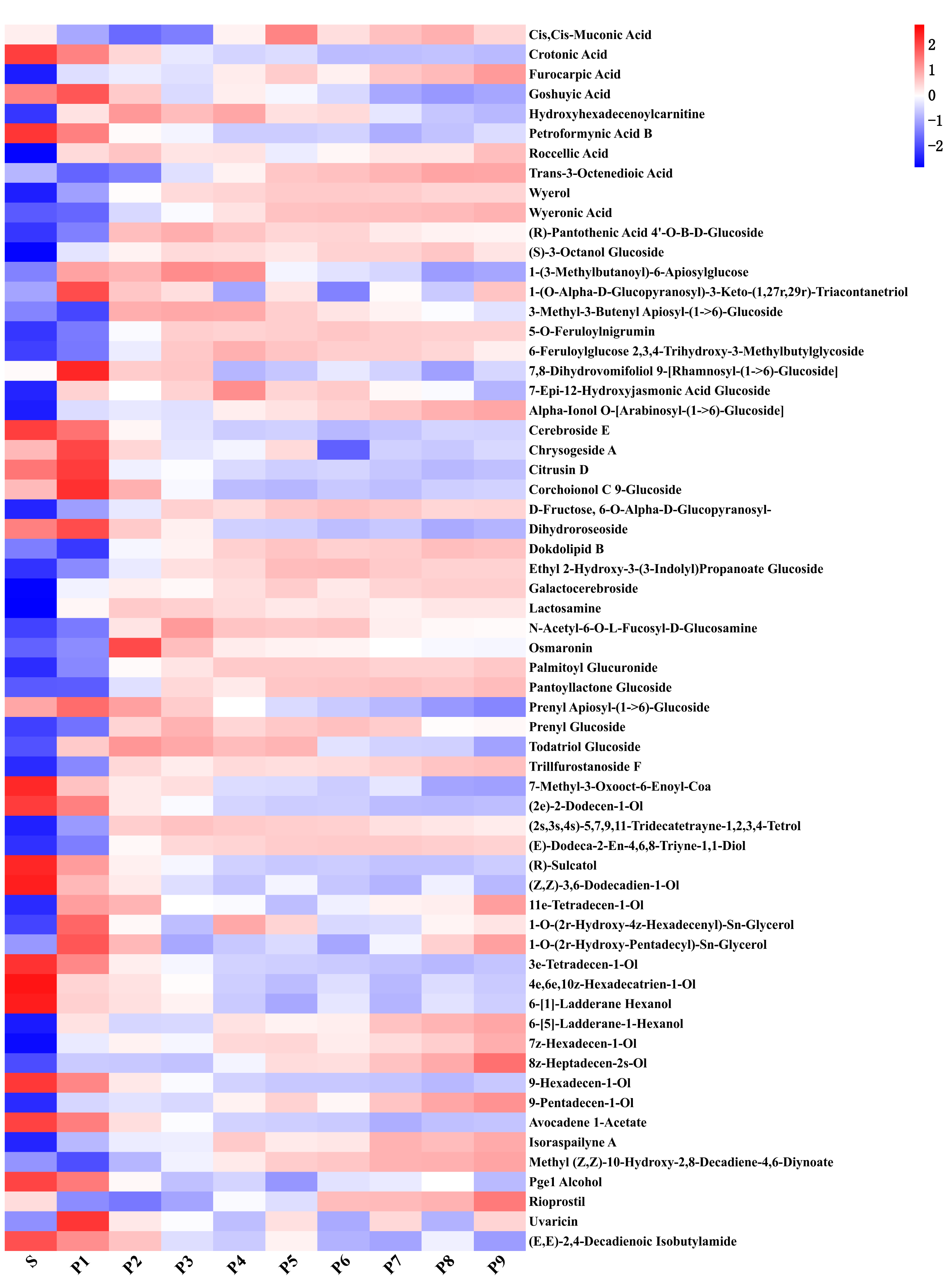

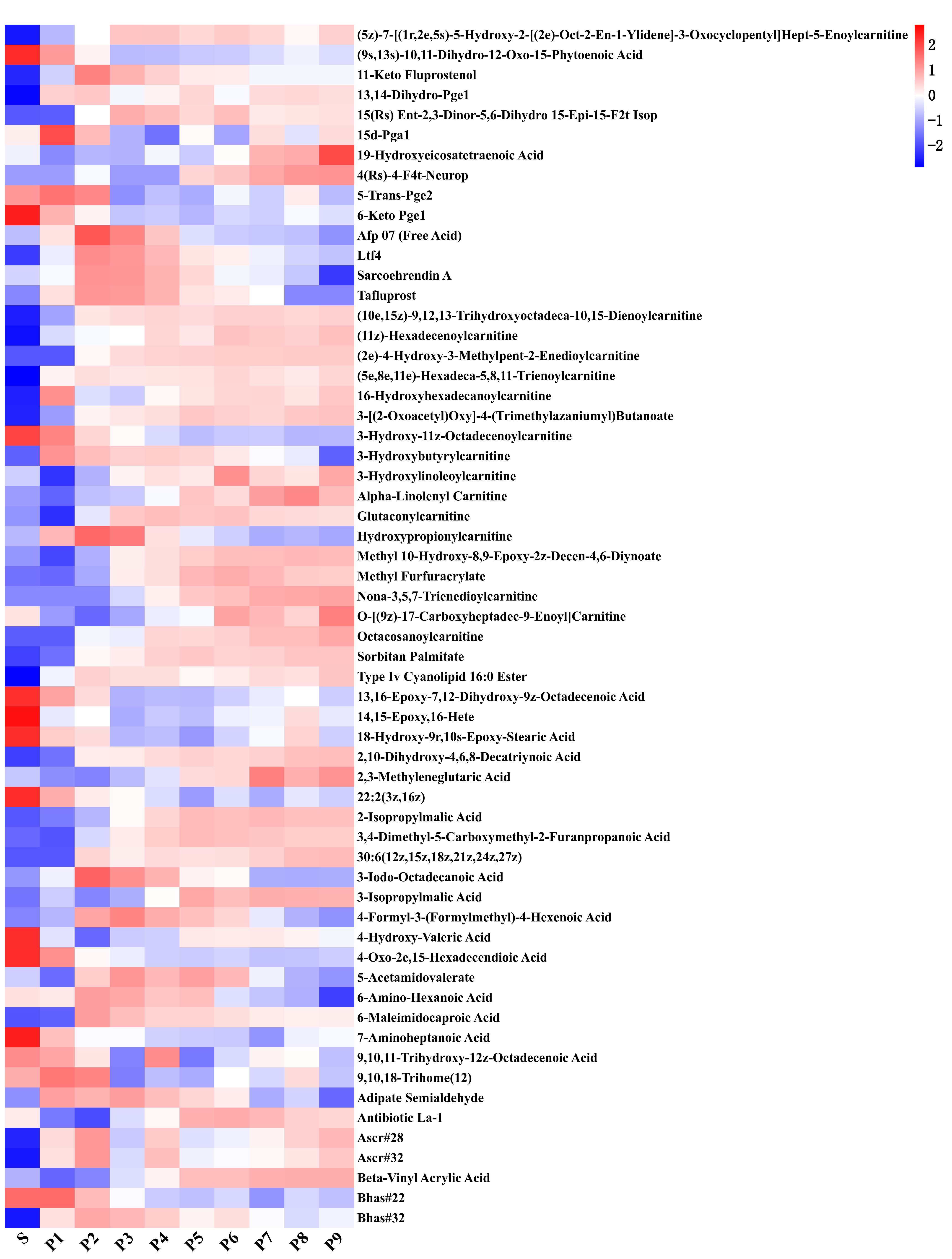

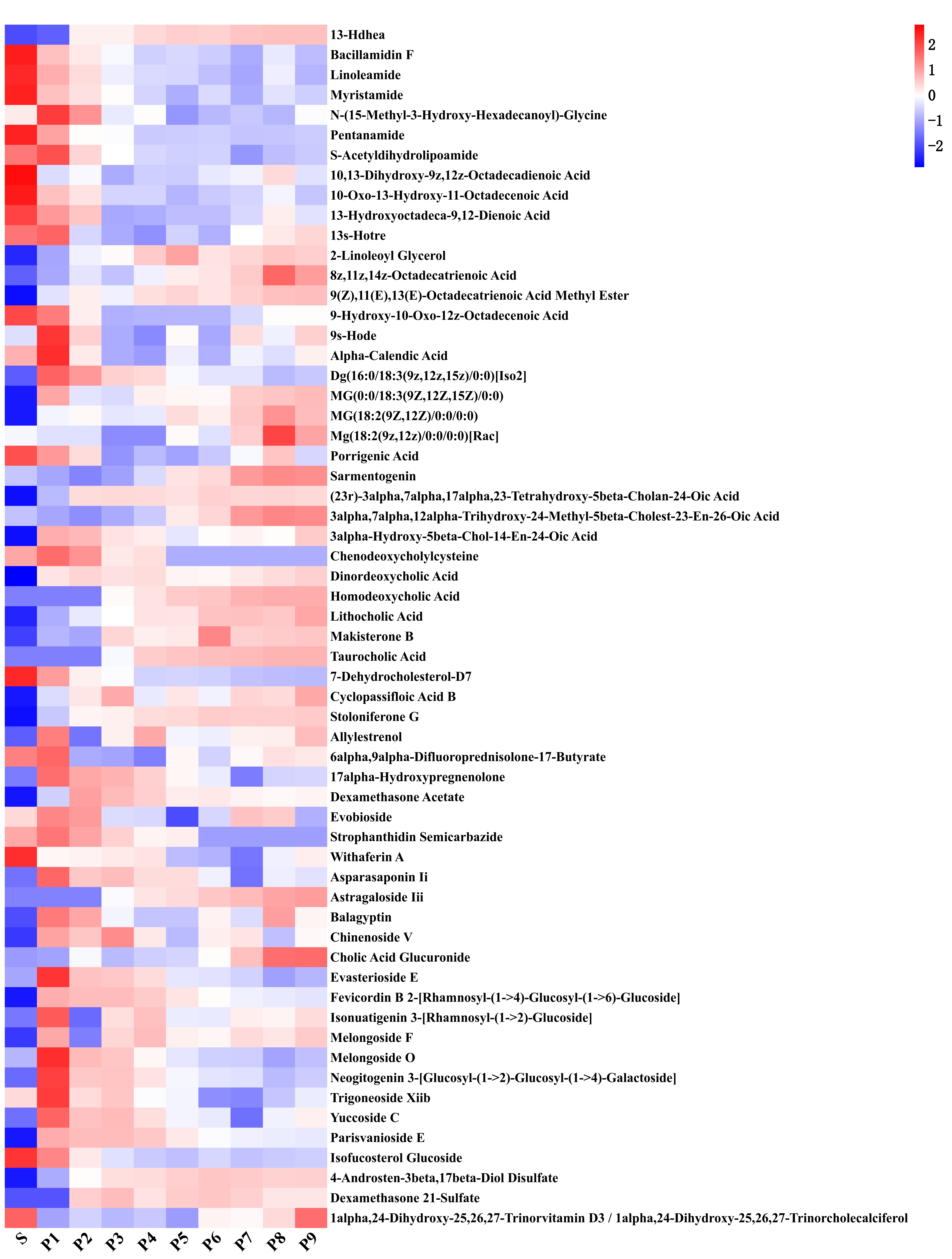


Steroids and steroid derivatives

**A**


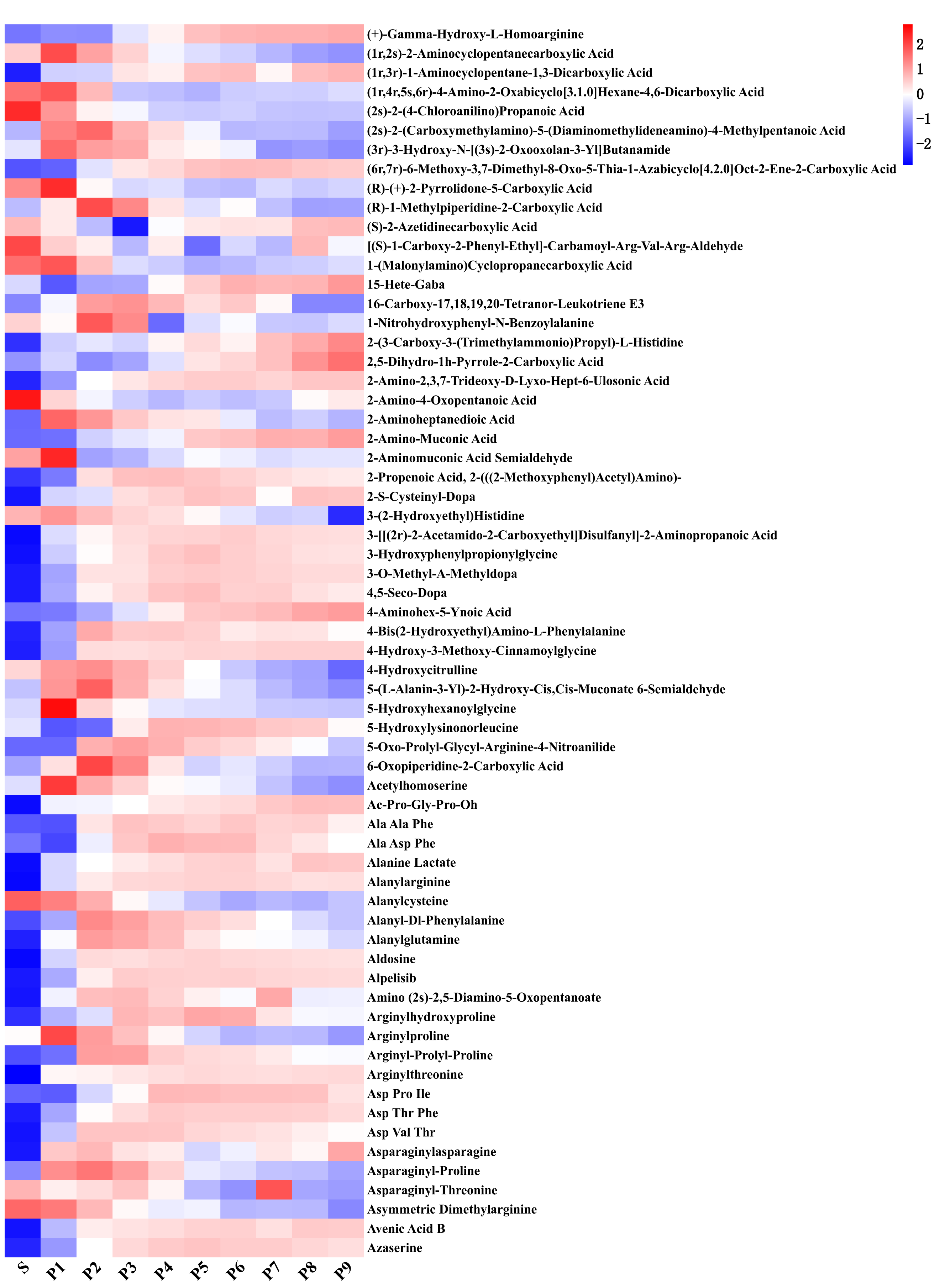

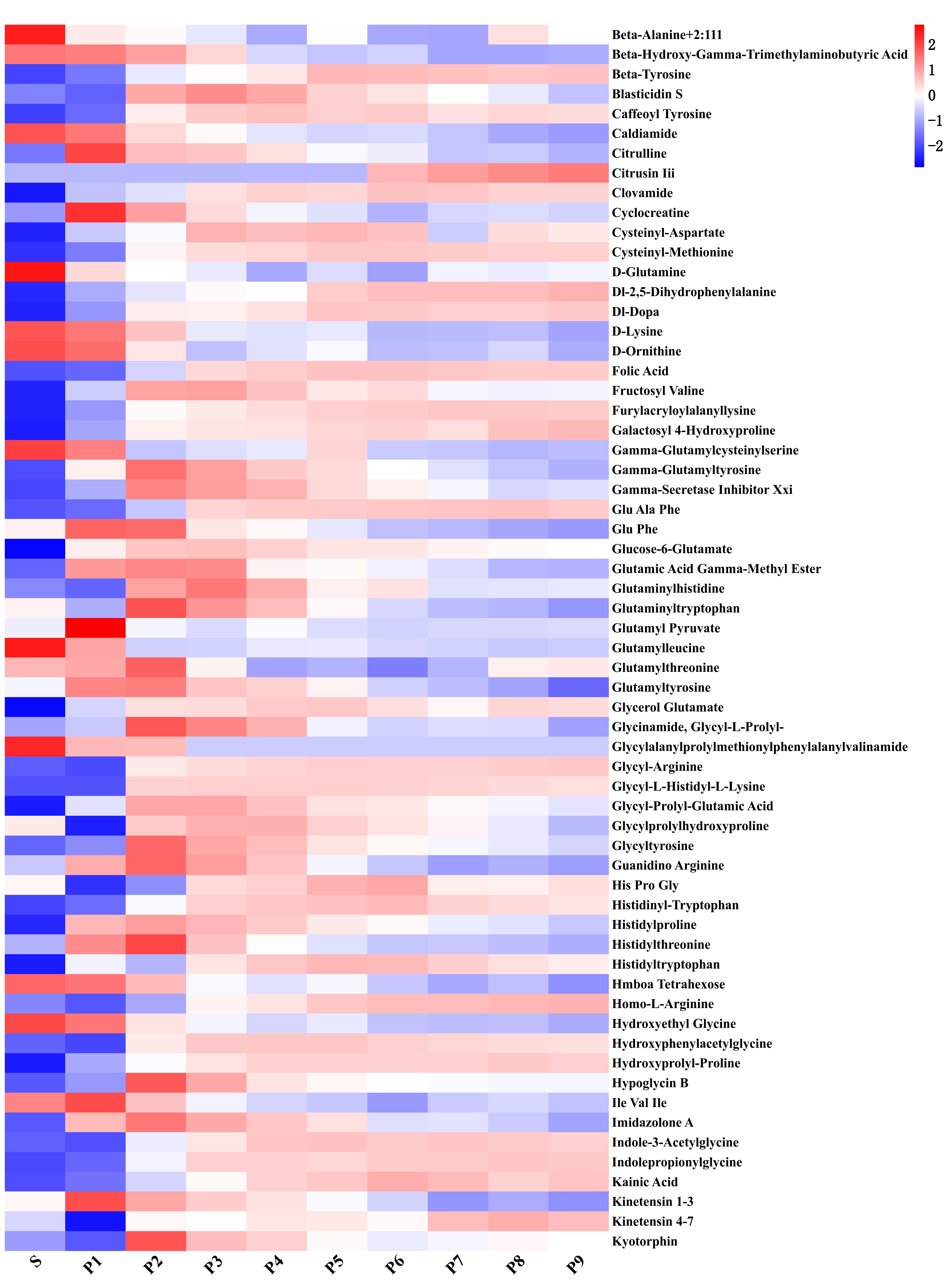

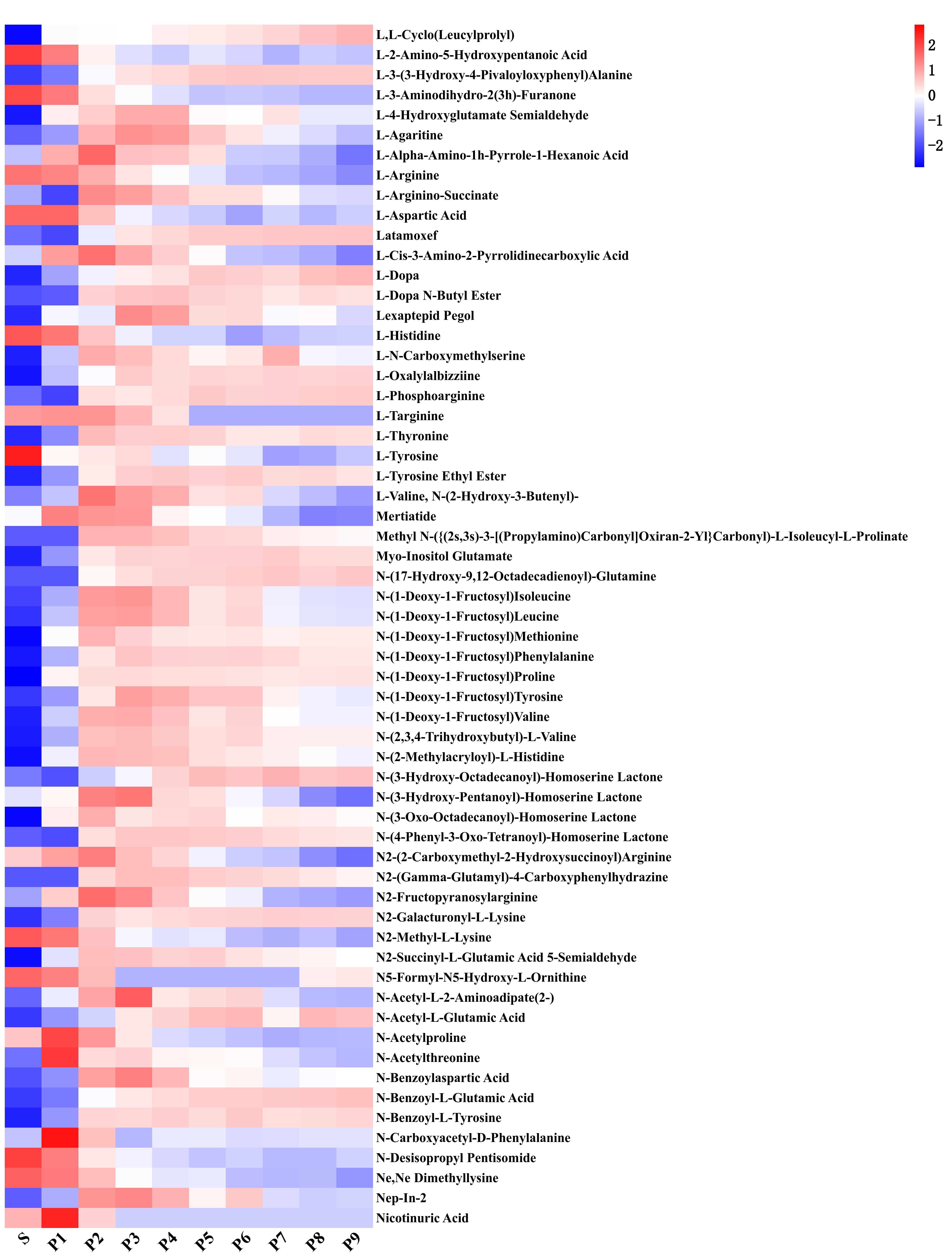

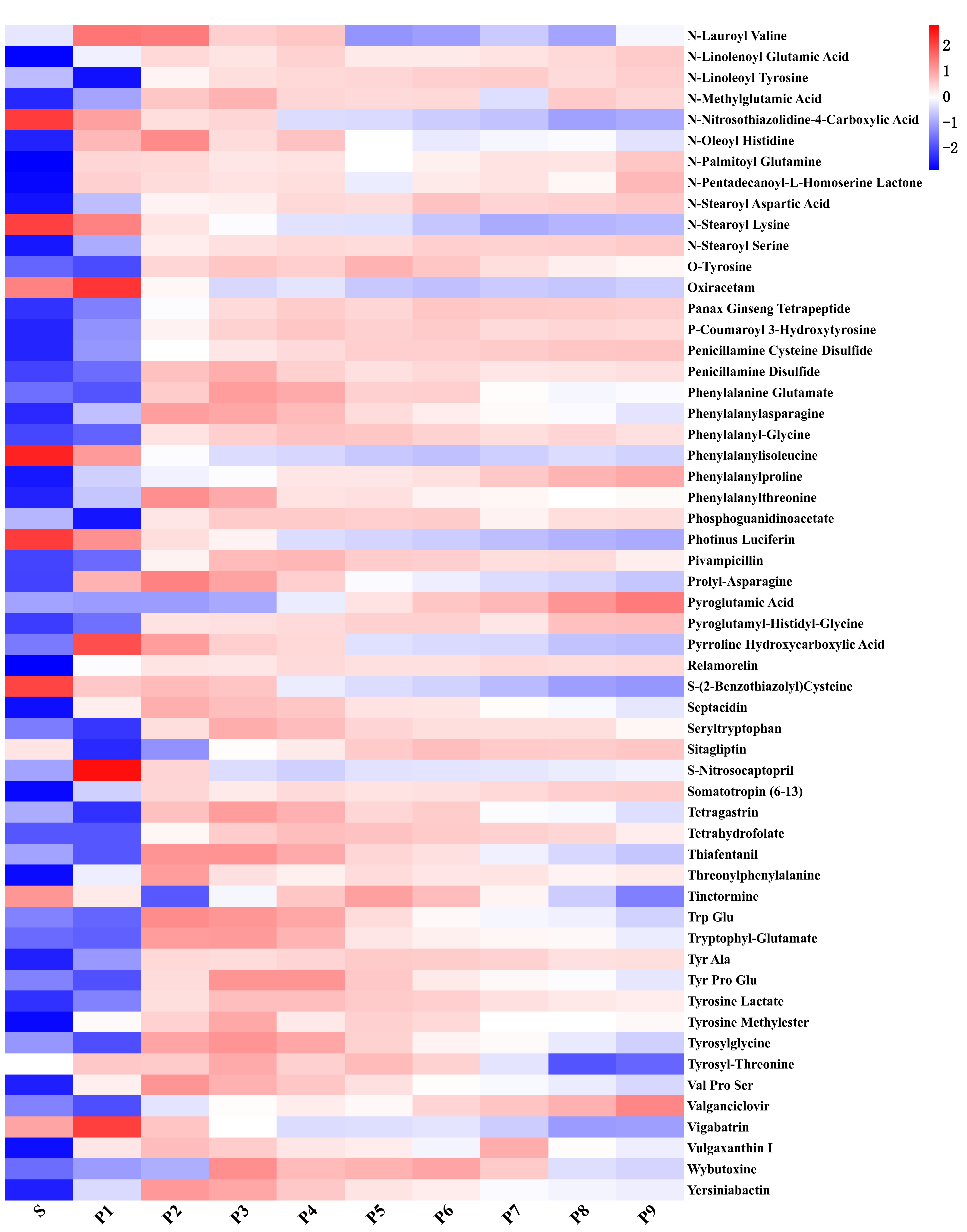


**B**


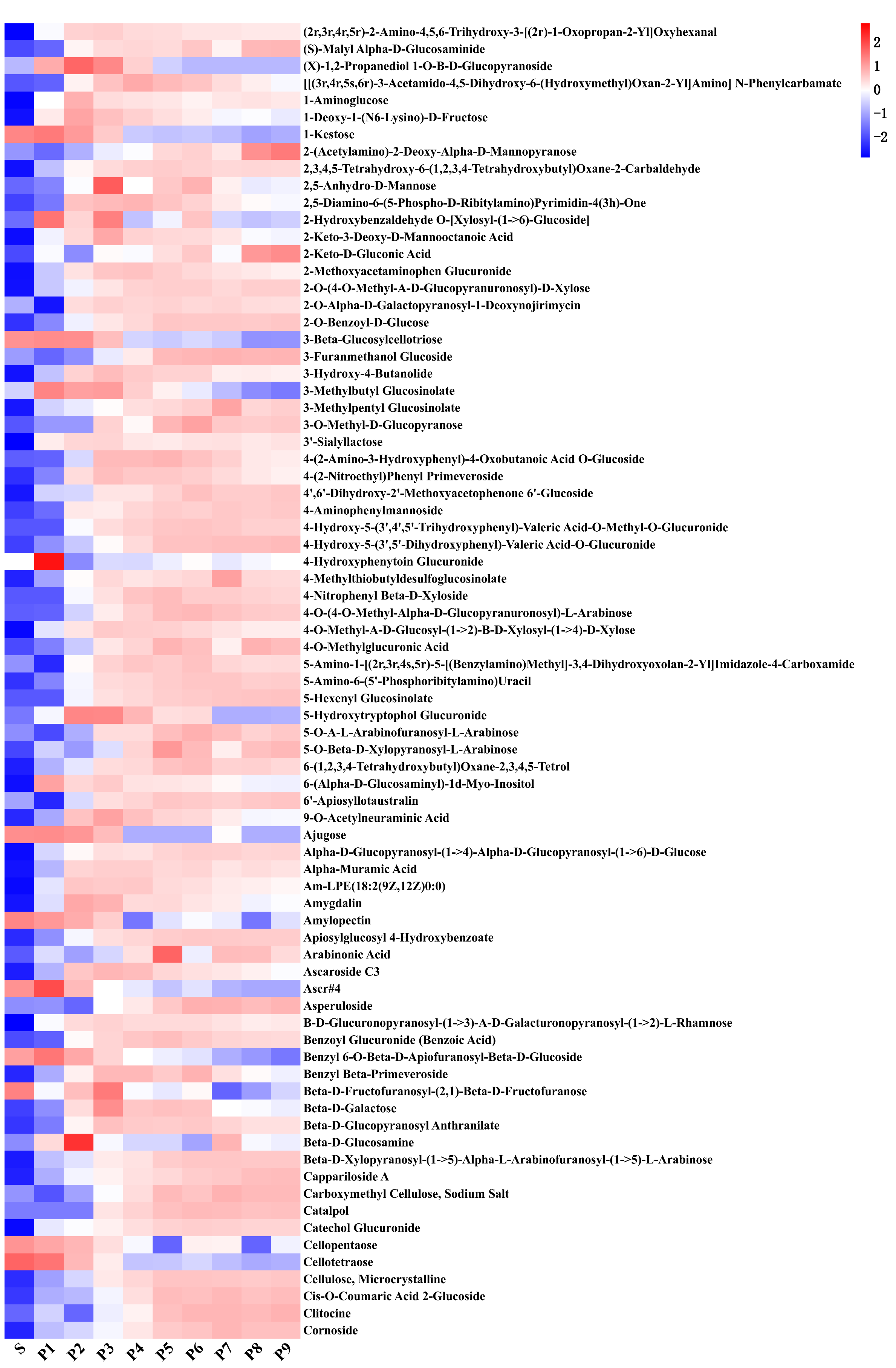

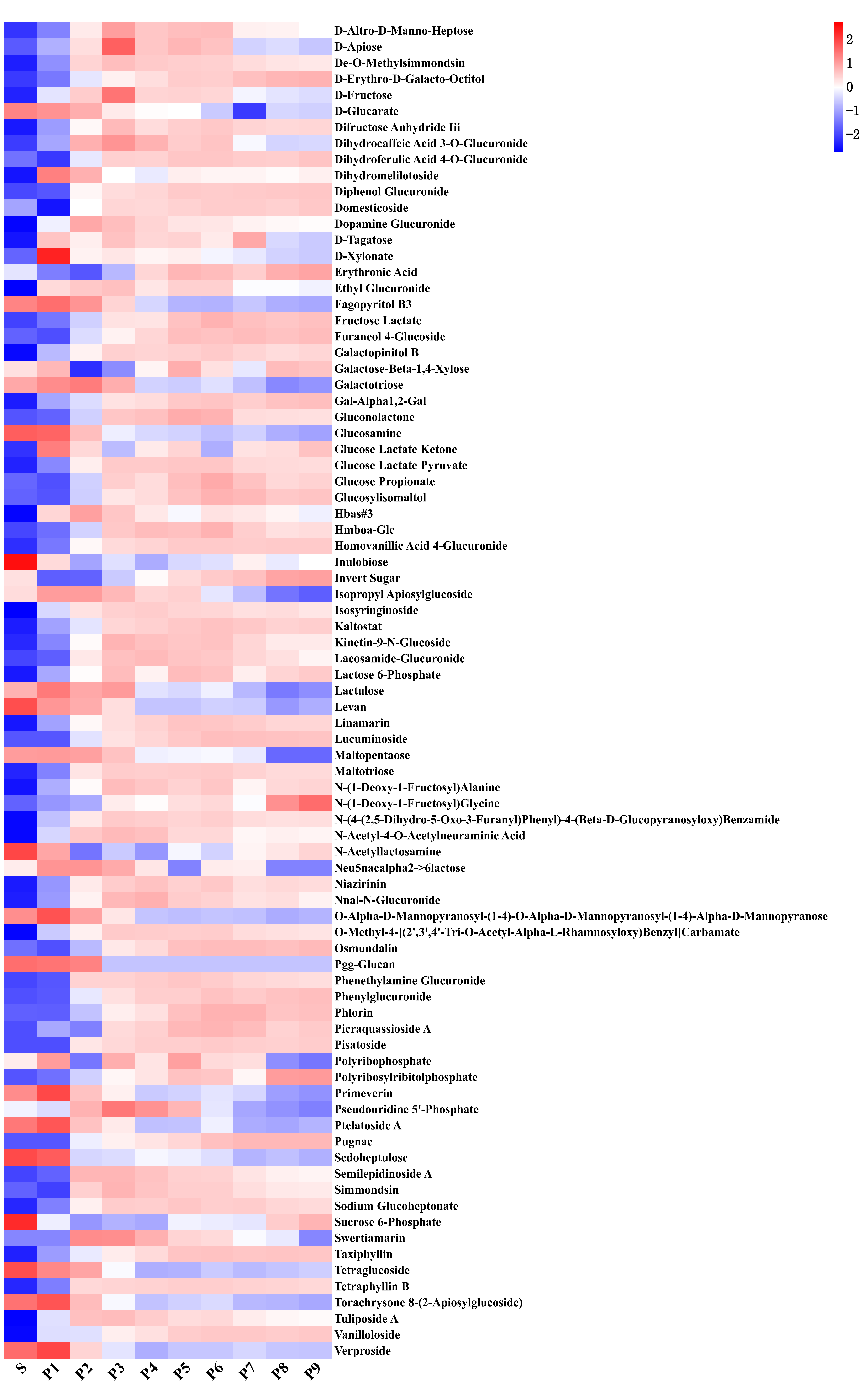


**C**


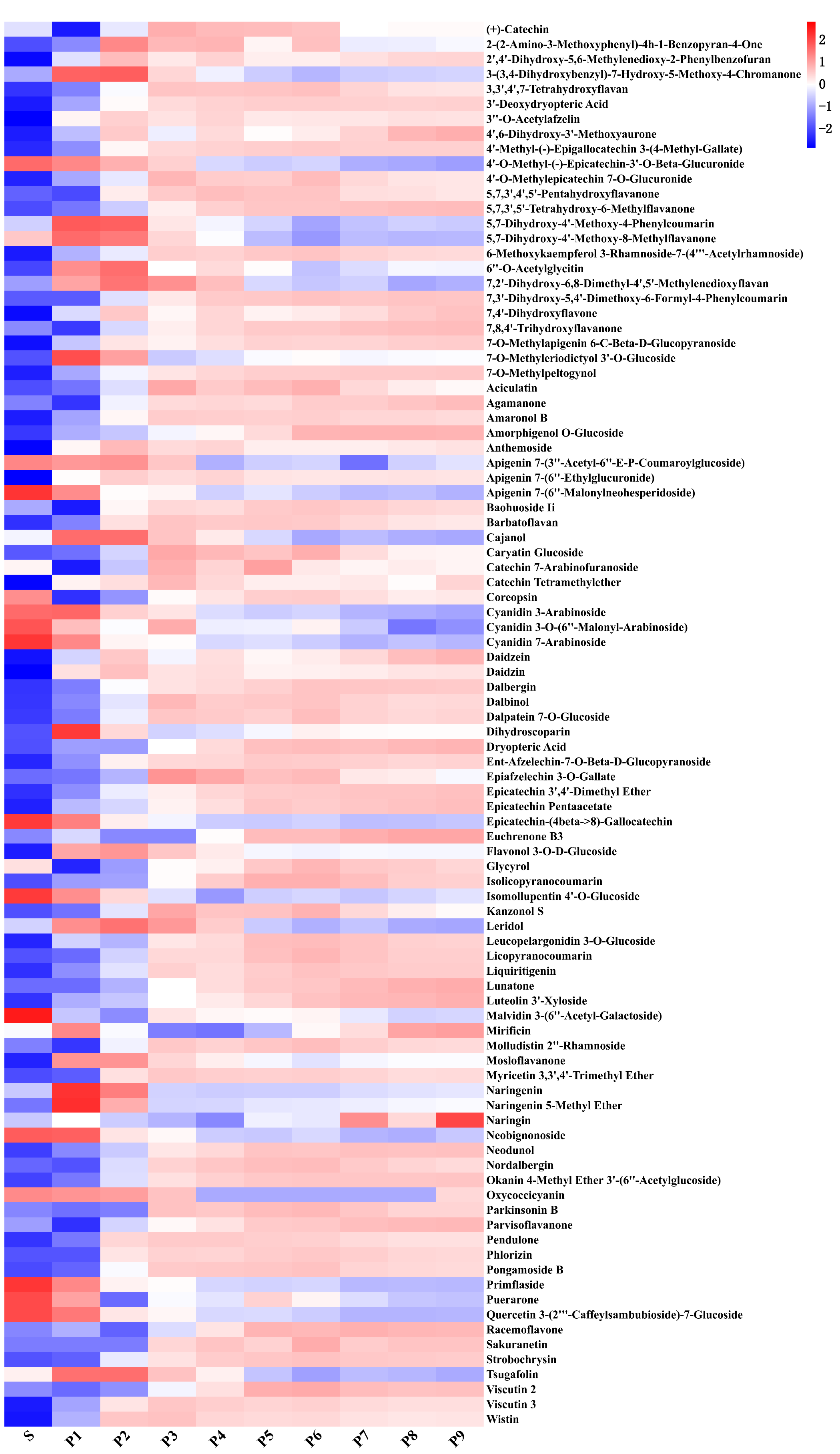


**E**


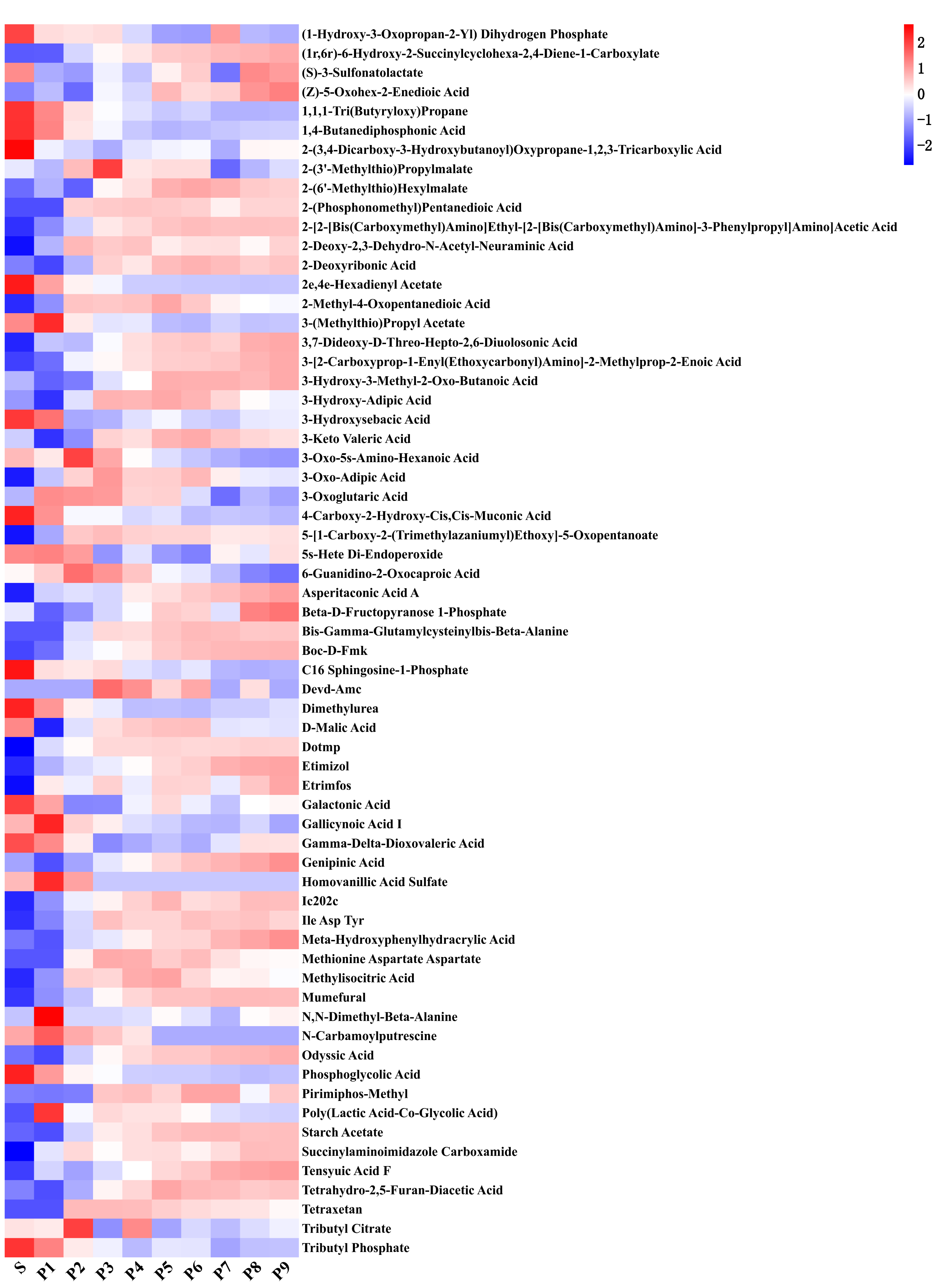


**D**


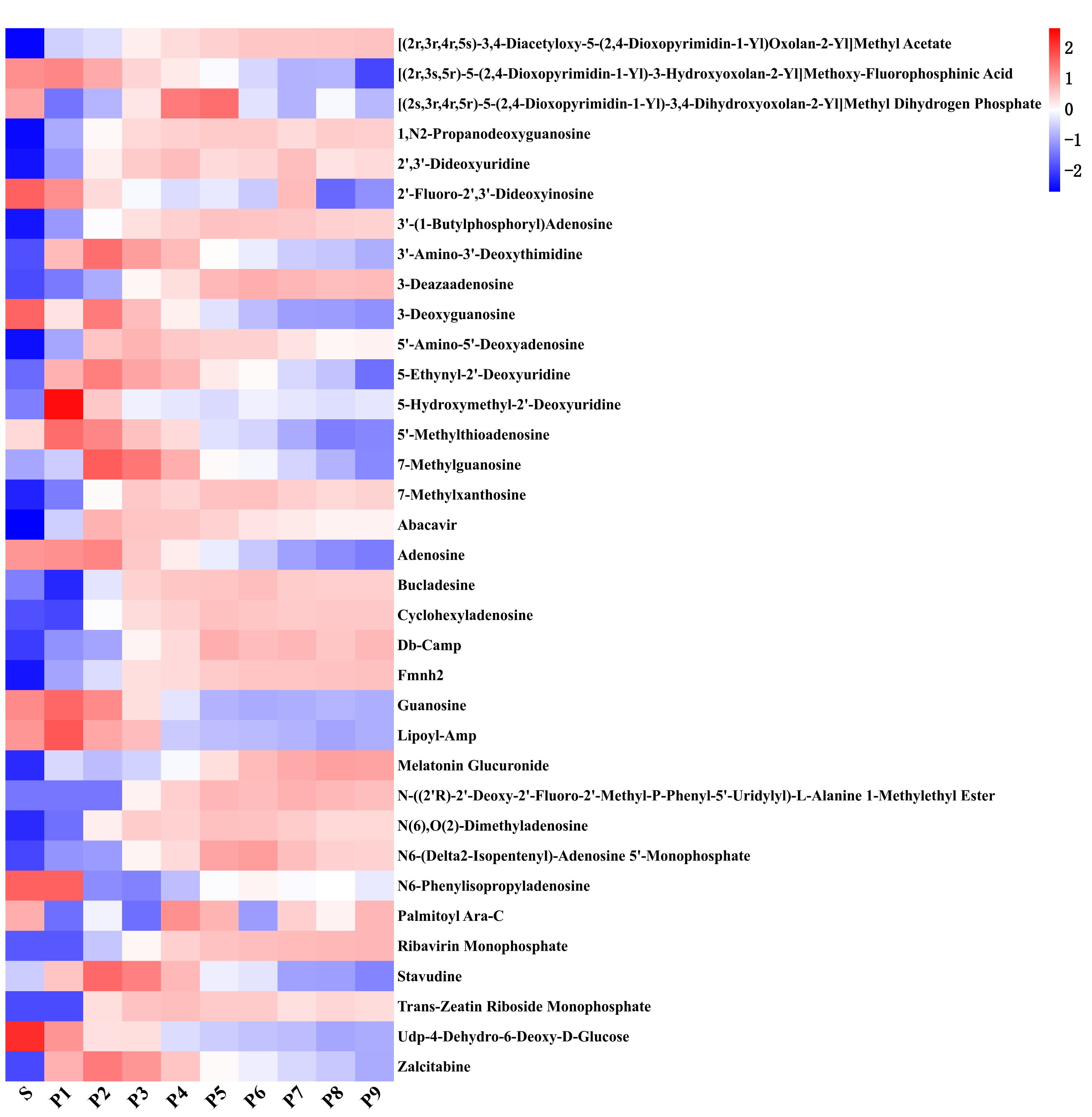


**F**


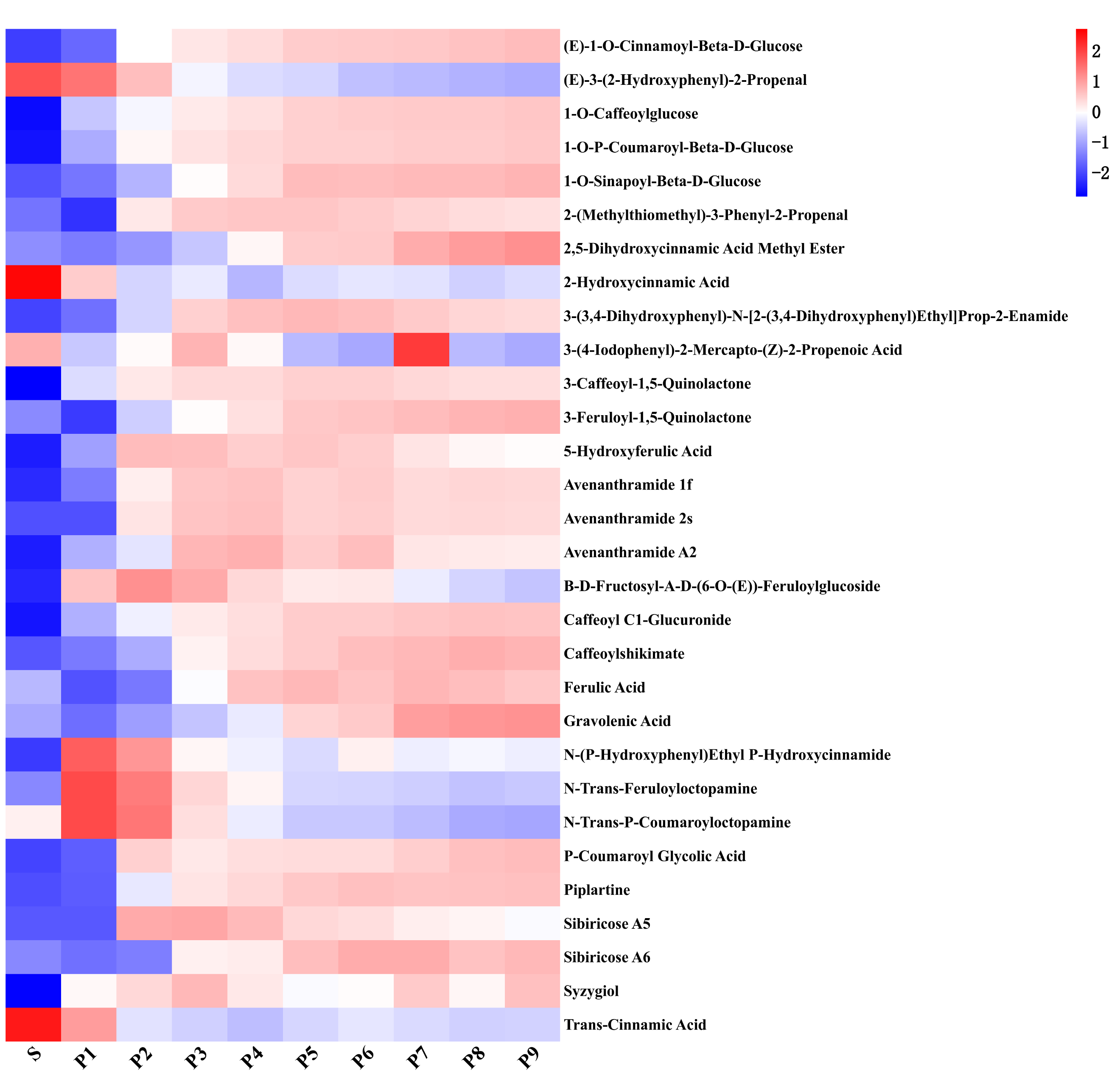


**G**


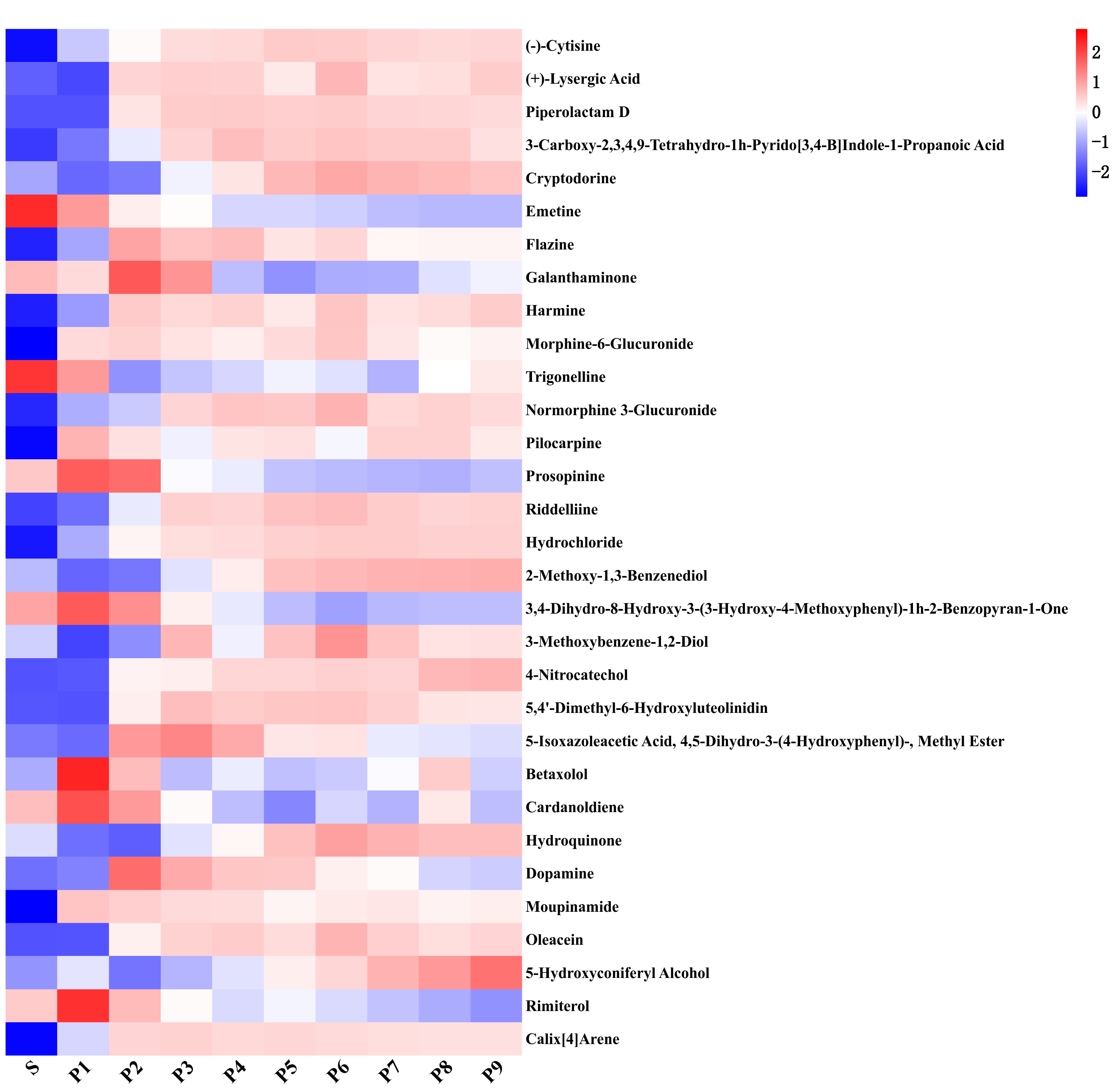


**H**

Alkaloids and derivatives

Phenols


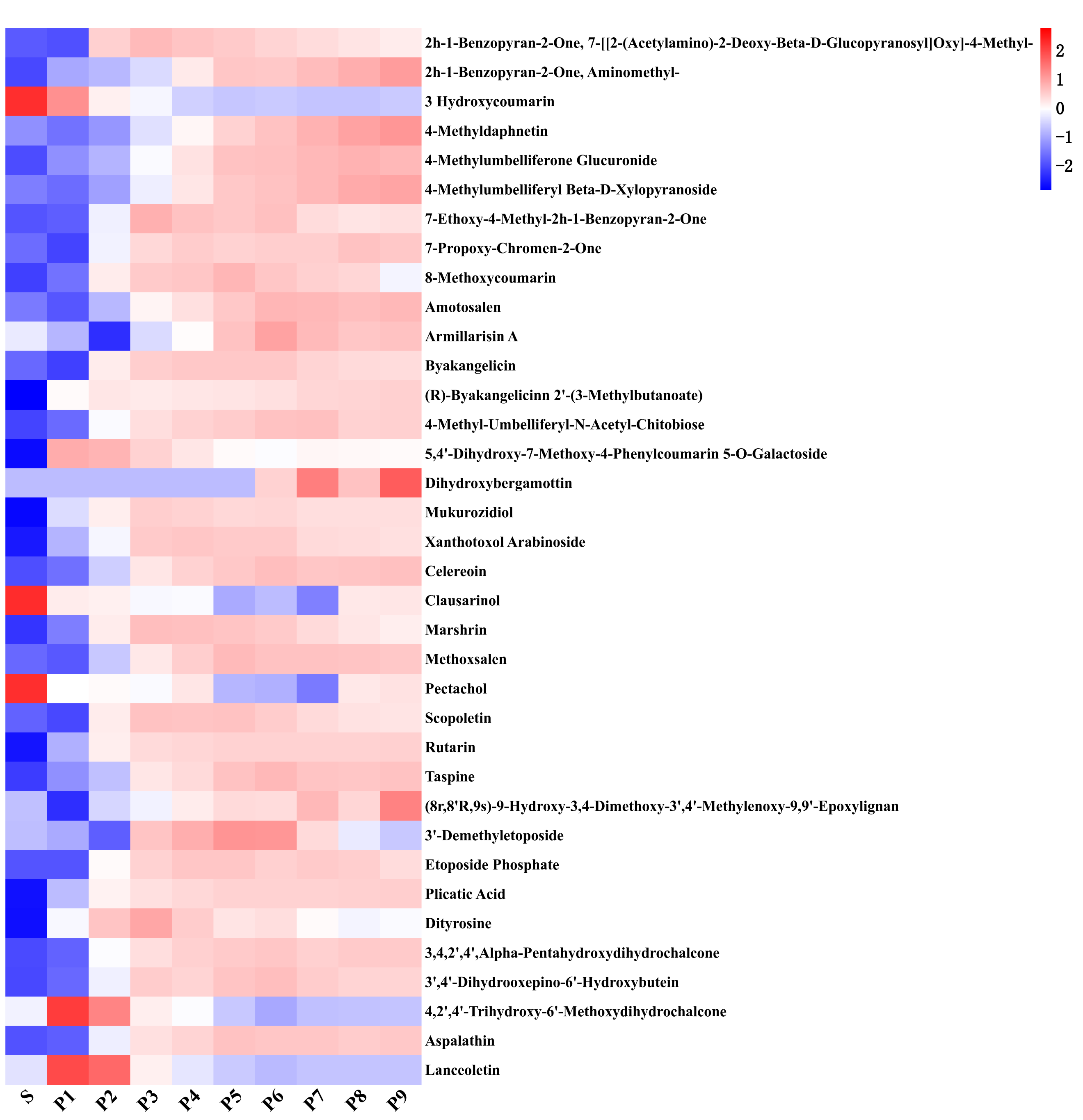


**I**

Coumarins, Isocoumarins and derivatives

Chalcones and dihydrochalcones

Lignans, neolignans and related compounds

**Figure S3.** Differential metabolite clustering heat map analysis: Fatty acyls, steroids and derivatives(A); Amino acids, peptides and analogues(B); Carbohydrates and carbohydrate conjugates(C); Organic acids and derivatives(D); Flavonoids and derivatives(E); Nucleosides, nucleotides, and analogues(F); Cinnamaldehydes, cinnamic acids and derivatives(G); Phenols, alkaloids and derivatives(H); Coumarins, lignans, chalcones and corresponding derivatives(I).
